# Supplementary material for: Reversing insufficient photothermal therapy-induced tumor relapse and metastasis by regulating cancer-associated fibroblasts
Source: Nat Commun. 2022 May 19;13:2794. doi: 10.1038/s41467-022-30306-7 (PMC9120472; doi:10.1038/s41467-022-30306-7)
Supplement: Supplementary file 1 — Supplementary Information [file 41467_2022_30306_MOESM1_ESM.pdf]

Reversing insufficient photothermal therapy-induced tumor relapse and metastasis by regulating cancer-associated fibroblasts

Xin Li[1,6], Tuying Yong[1,2,3,4,6,*], Zhaohan Wei[1], Nana Bie[1], Xiaoqiong Zhang[1], Guiting Zhan[1], Jianye Li[1], Jiaqi Qin[1], Jingjing Yu[5], Bixiang Zhang[5], Lu Gan[1,2,3,4,*], Xiangliang Yang[1,2,3,4,*]

[1]National Engineering Research Center for Nanomedicine, College of Life Science and Technology, Huazhong University of Science and Technology, Wuhan 430074, China

[2]Key Laboratory of Molecular Biophysics of the Ministry of Education, College of Life Science and Technology, Huazhong University of Science and Technology, Wuhan 430074, China

[3]Hubei Key Laboratory of Bioinorganic Chemistry and Materia Medica, Huazhong University of Science and Technology, Wuhan 430074, China

[4]Hubei Engineering Research Center for Biomaterials and Medical Protective Materials, Huazhong University of Science and Technology, Wuhan 430074, China

[5]Hepatic Surgery Center, Tongji Hospital, Tongji Medical College, Huazhong University of Science and Technology, Wuhan 430030, China.

[6]These authors contributed equally.

*email: lugan@mail.hust.edu.cn; yongty2018@mail.hust.edu.cn; yangxl@mail.hust.edu.cn.

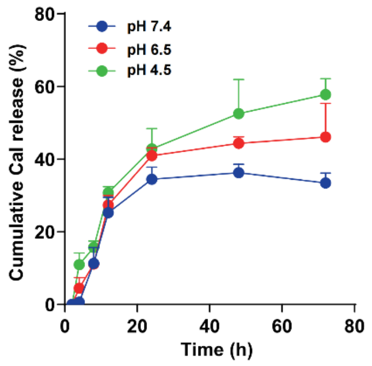

Supplementary Fig. 1 In vitro Cal release of Cal/ICG@MPs.

In vitro Cal release profiles of Cal/ICG@MPs in PBS containing 10% FBS at different pH values by HPLC analysis. Data are presented as means ± s.d. (n = 3 independent samples). Source data are provided as a Source Data file.

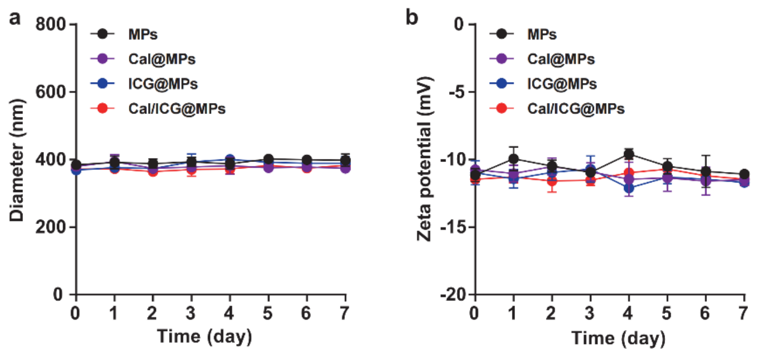

Supplementary Fig. 2 Stability of Cal/ICG@MPs.

a, b Diameters (**a**) and zeta potentials (**b**) of MPs, Cal@MPs, ICG@MPs and Cal/ICG@MPs after incubation in PBS for different time intervals. Data are presented as mean ± s.d. (n = 4 independent samples). Source data are provided as a Source Data file.

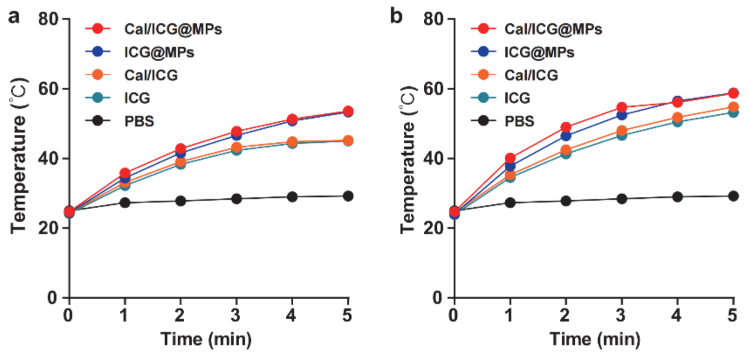

Supplementary Fig. 3 In vitro photothermal effects of Cal/ICG@MPs.

a, b Temperature curves of PBS, ICG, Cal/ICG, ICG@MPs and Cal/ICG@MPs at the ICG concentration of 12.5 μg mL^{-1} (**a**) or 25 μg mL^{-1} (**b**) upon 808 nm laser irradiation (1 W cm^{-2}, 5 min). Data are representative of three independent samples. Source data are provided as a Source Data file.

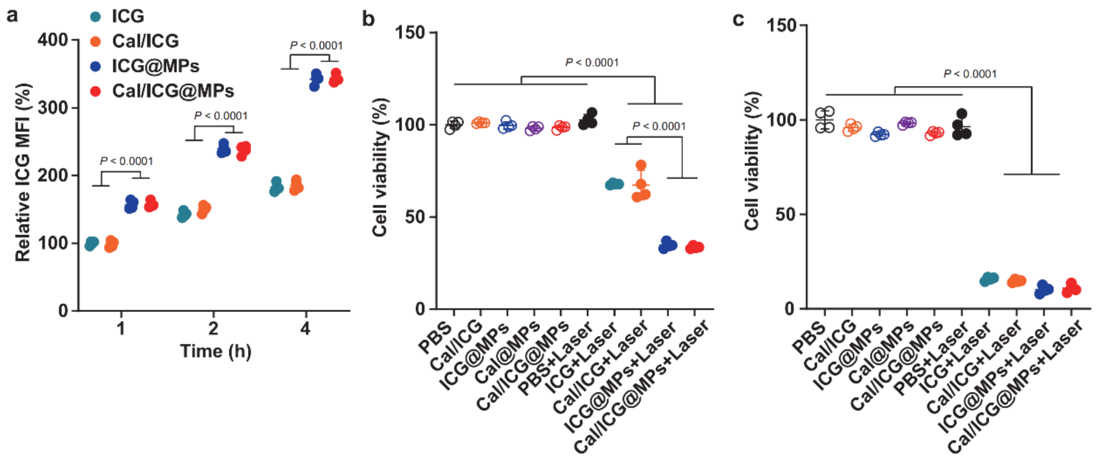

Supplementary Fig. 4 Cal/ICG@MPs-triggered cytotoxicity against H22 cells upon 808 nm laser irradiation.

a Relative ICG MFI in H22 cells after treatment with ICG, Cal/ICG, ICG@MPs or Cal/ICG@MPs derived from H22 cells at the ICG concentration of 0.5 μg mL^{-1} for different time intervals. Data are presented as means ± s.d. (n = 4 biologically independent samples; two-way ANOVA followed by Tukey's multiple comparisons post-test). **b, c** Cell viability of H22 cells after treatment with PBS, ICG, Cal/ICG, ICG@MPs, Cal@MPs or Cal/ICG@MPs at the ICG concentration of 2 μg mL^{-1} (**b**) or 8 μg mL^{-1} (**c**) for 4 h in the presence or absence of 808 nm laser irradiation (1 W cm^{-2}, 5 min). Data are presented as means ± s.d. (n = 4 biologically independent samples; one-way ANOVA followed by Tukey's HSD post-hoc test). Source data are provided as a Source Data file.

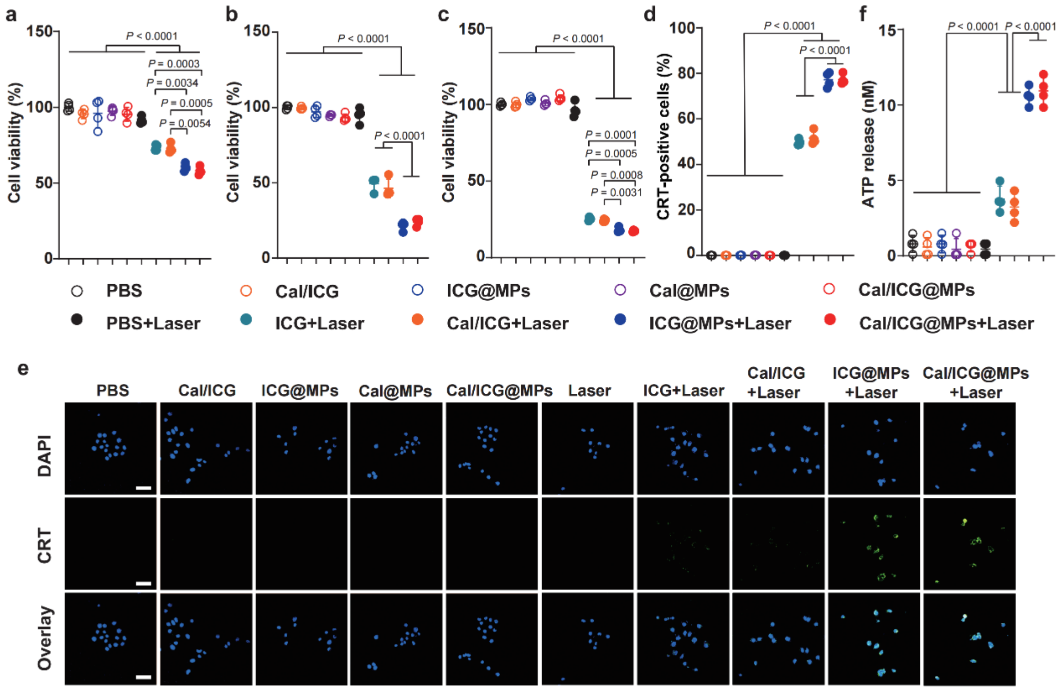

Supplementary Fig. 5 Cal/ICG@MPs-triggered cell cytotoxicity and ICD effects in 4T1 cells upon 808 nm laser irradiation.

a-c Cell viability of 4T1 cells after treatment with PBS, ICG, Cal/ICG, ICG@MPs, Cal@MPs or Cal/ICG@MPs derived from 4T1 cells at the ICG concentration of 2 μg mL^{-1} (**a**), 4 μg mL^{-1} (**b**) or 8 μg mL^{-1} (**c**) for 4 h in the presence or absence of 808 nm laser irradiation (1 W cm^{-2}, 5 min). Data are presented as means ± s.d. (n = 4 biologically independent samples; one-way ANOVA followed by Tukey's HSD post-hoc test). **d, e** CRT exposure on 4T1 cells after treatment with PBS, ICG, Cal/ICG, ICG@MPs, Cal@MPs or Cal/ICG@MPs at the ICG concentration of 4 μg mL^{-1} and Cal concentration of 60 ng mL^{-1} for 4 h in the presence or absence of 808 nm laser irradiation (1 W cm^{-2}, 5min) by flow cytometry (**d**) and confocal microscopy (**e**). Data are presented as means ± s.d. (n = 4 biologically independent samples; one-way ANOVA followed by Tukey's HSD post-hoc test) for **d**; Images are representative of four biologically independent samples. Scale bars: 50 μm for **e**. **f** ATP extracellular secretion from 4T1 cells after treatment indicated in (**d**). Data are presented as means ± s.d. (n = 4 biologically independent samples; one-way ANOVA followed by Tukey's HSD post-hoc test). Source data are provided as a Source Data file.

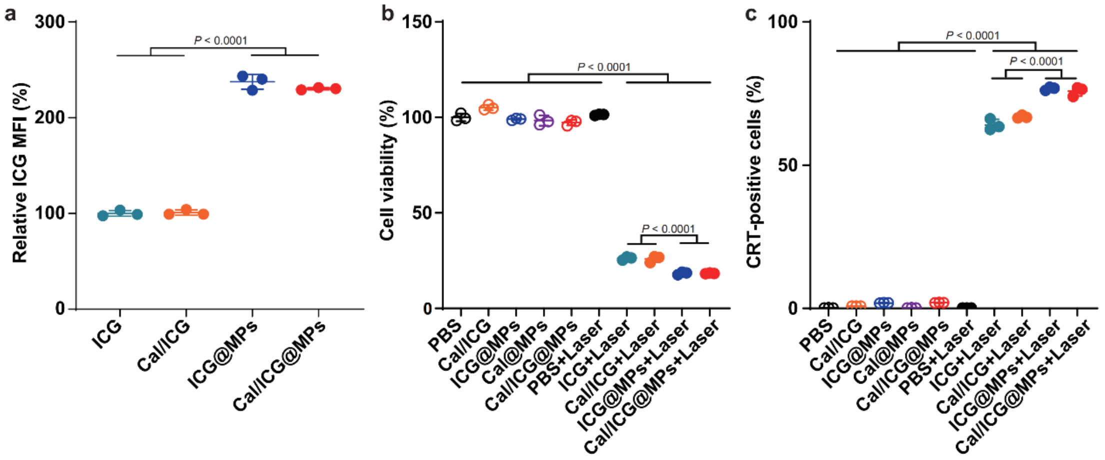

Supplementary Fig. 6 Cal/ICG@MPs-triggered cell cytotoxicity and ICD effects in HepG2 cells upon 808 nm laser irradiation.

a Relative ICG MFI in HepG2 cells after treatment with ICG, Cal/ICG, ICG@MPs or Cal/ICG@MPs derived from HepG2 cells at the ICG concentration of 0.5 μg mL^{-1} for 4 h. Data are presented as means ± s.d. (n = 3 biologically independent samples; one-way ANOVA followed by Tukey's HSD post-hoc test). **b** Cell viability of HepG2 cells after treatment with PBS, ICG, Cal/ICG, ICG@MPs, Cal@MPs or Cal/ICG@MPs at the ICG concentration of 4 μg mL^{-1} and Cal concentration of 60 ng mL^{-1} for 4 h in the presence or absence of 808 nm laser irradiation (1 W cm^{-2}, 5 min). Data are presented as means ± s.d. (n = 3 biologically independent samples; one-way ANOVA followed by Tukey's HSD post-hoc test). **c** CRT exposure on HepG2 cells after treatment indicated in (**b**) by flow cytometry. Data are presented as means ± s.d. (n = 3 biologically independent samples; one-way ANOVA followed by Tukey's HSD post-hoc test). Source data are provided as a Source Data file.

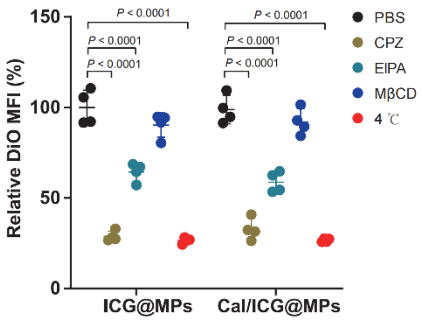

Supplementary Fig. 7 Endocytic pathway of ICG@MPs and Cal/ICG@MPs.

Intracellular DiO fluorescence intensity in H22 cells after treatment with DiO-labeled ICG@MPs or Cal/ICG@MPs derived from H22 cells at the concentration of 10 μg protein mL^{-1} in the presence or absence of 1.25 mg mL^{-1} methyl-β-cyclodextrin (MβCD, an inhibitor of caveolin), 12.5 μg mL^{-1} 5-(*N*-ethyl-*N*-isopropyl) amiloride (EIPA, an inhibitor of macropinocytosis) or 5 μg mL^{-1} chlorpromazine (CPZ, an inhibitor of clathrin) at 37 or 4 °C for 4 h by flow cytometry. Data are presented as means ± s.d. (n = 4 biologically independent samples; two-way ANOVA followed by Tukey's multiple comparisons post-test). Source data are provided as a Source Data file.

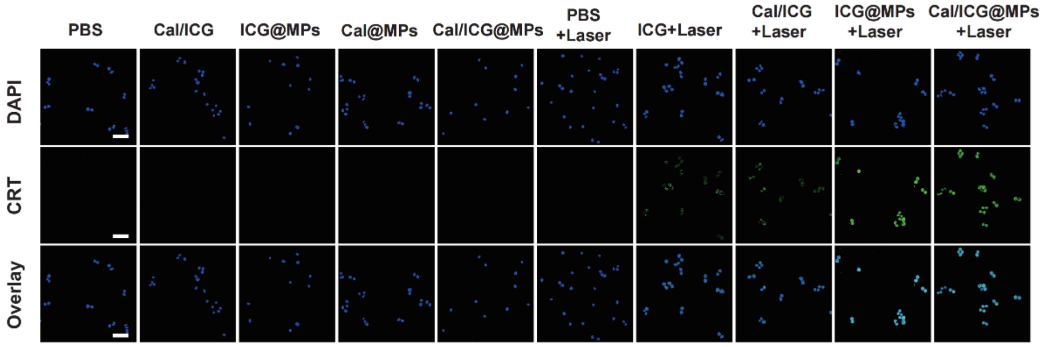

Supplementary Fig. 8 CRT exposure on H22 cells after treatment with Cal/ICG@MPs upon 808 nm laser irradiation.

CRT exposure on H22 cells after treatment with PBS, ICG, Cal/ICG, ICG@MPs, Cal@MPs or Cal/ICG@MPs derived from H22 cells at the ICG concentration of 4 μg mL^{-1} and Cal concentration of 60 ng mL^{-1} for 4 h in the presence or absence of 808 nm laser irradiation (1 W cm^{-2}, 5 min) by confocal microscopy. Images are representative of four biologically independent samples. Scale bars: 50 μm.

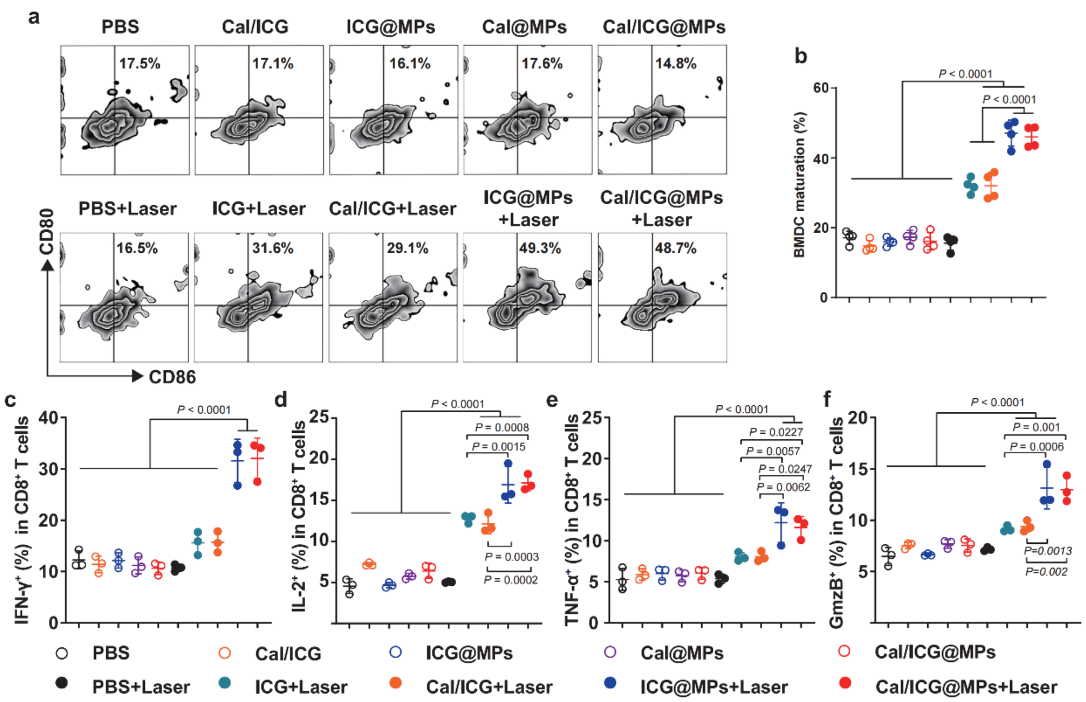

Supplementary Fig. 9 In vitro DC maturation and T cell activation triggered by Cal/ICG@MPs with 808 nm laser irradiation-treated 4T1 cells.

a, b Representative flow cytometric plots (**a**) and percentages (**b**) of matured BMDCs ($CD11c^+CD80^+CD86^+$) after immature BMDCs were co-cultured with the cell supernatants from 4T1 cells treated with PBS, ICG, Cal/ICG, ICG@MPs, Cal@MPs or Cal/ICG@MPs derived from 4T1 cells at the ICG concentration of 4 μg mL^{-1} and Cal concentration of 60 ng mL^{-1} (4 h) in the presence or absence of 808 nm laser irradiation (1 W cm^{-2}, 5 min) for 24 h. Data are presented as means ± s.d. (n = 4 biologically independent samples; one-way ANOVA followed by Tukey's HSD post-hoc test). **c-f** Percentages of IFN-γ^+ (**c**), IL-2^+ (**d**), TNF-α^+ (**e**) and $GzmB^+$ cells (**f**) in $CD8^+$ T cells after $CD3^+$ T cells were incubated with the above matured BMDCs for 5 days by flow cytometry. Data are presented as means ± s.d. (n = 3 biologically independent samples; one-way ANOVA followed by Tukey's HSD post-hoc test). Source data are provided as a Source Data file.

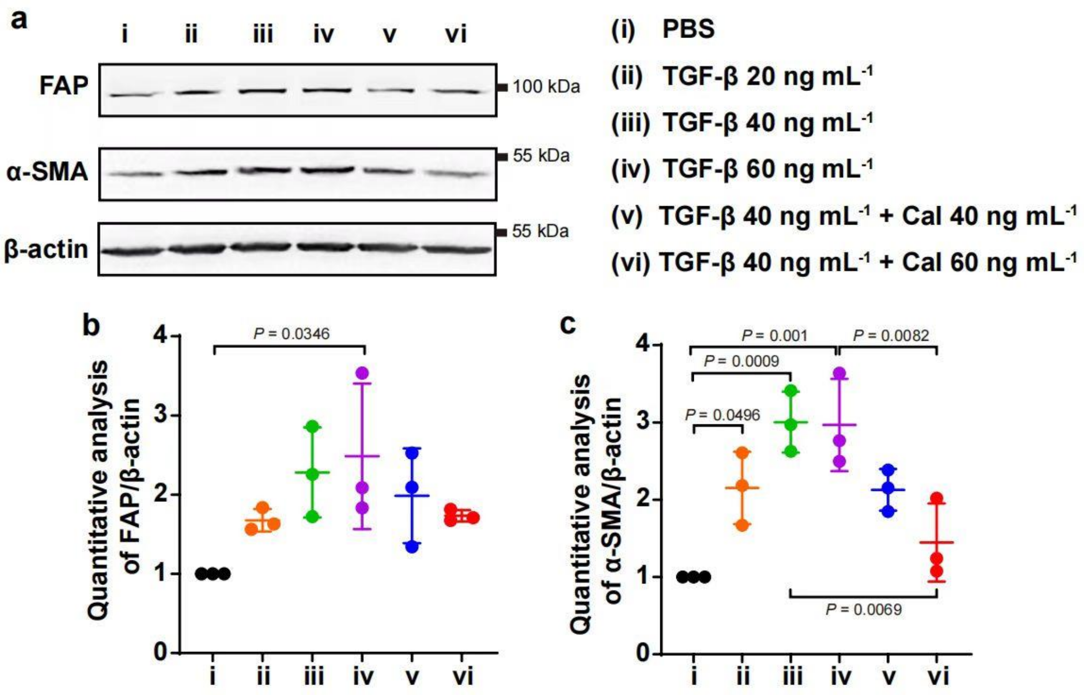

Supplementary Fig. 10 Myofibroblasts induction by TGF-β and regulation by Cal.

a FAP and α-SMA expression was detected by western blot after skin fibroblasts were treated with different concentrations (20, 40 and 60 ng mL^{-1}) of TGF-β for 24 h and then deactivated with different concentrations (40 and 60 ng mL^{-1}) of Cal for 48 h. Images are representative of three independent samples. **b, c** Quantitative analysis of the normalized FAP (**b**) and α-SMA (**c**) protein expression using Image J software. Data are presented as means ± s.d. (n = 3 biologically independent samples; one-way ANOVA followed by Tukey's HSD post-hoc test). Source data are provided as a Source Data file.

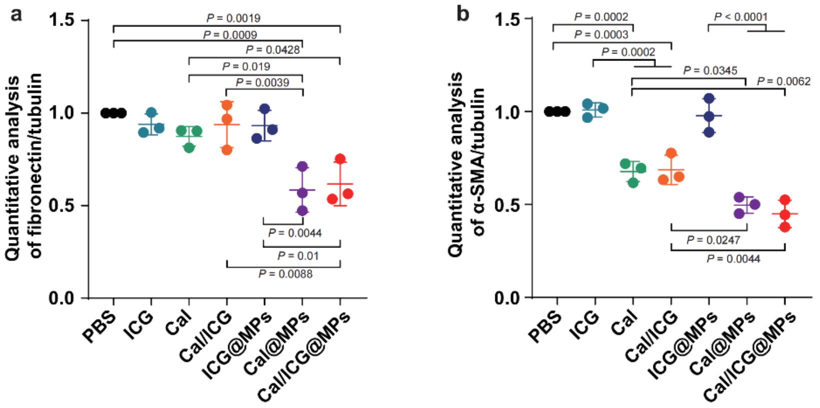

Supplementary Fig. 11 Quantitative analysis of fibronectin and α-SMA levels from western blot assay.

a, b Quantitative analysis of the normalized protein expression of fibronectin (**a**) and α-SMA (**b**) in myofibroblasts as indicated in Fig. **4a**. Data are presented as means ± s.d. (n = 3 biologically independent samples; one-way ANOVA followed by Tukey's HSD post-hoc test). Source data are provided as a Source Data file.

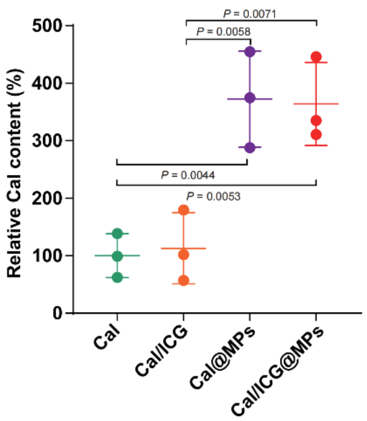

Supplementary Fig. 12 Cellular uptake of Cal/ICG@MPs by myofibroblasts.

Relative Cal content in myofibroblasts after treatment with Cal, Cal/ICG, Cal@MPs or Cal/ICG@MPs derived from H22 cells at the Cal concentration of 60 ng mL^{-1} for 4 h by HPLC. Data are presented as means ± s.d. (n=3 biologically independent samples; one-way ANOVA followed by Tukey's HSD post-hoc test). Source data are provided as a Source Data file.

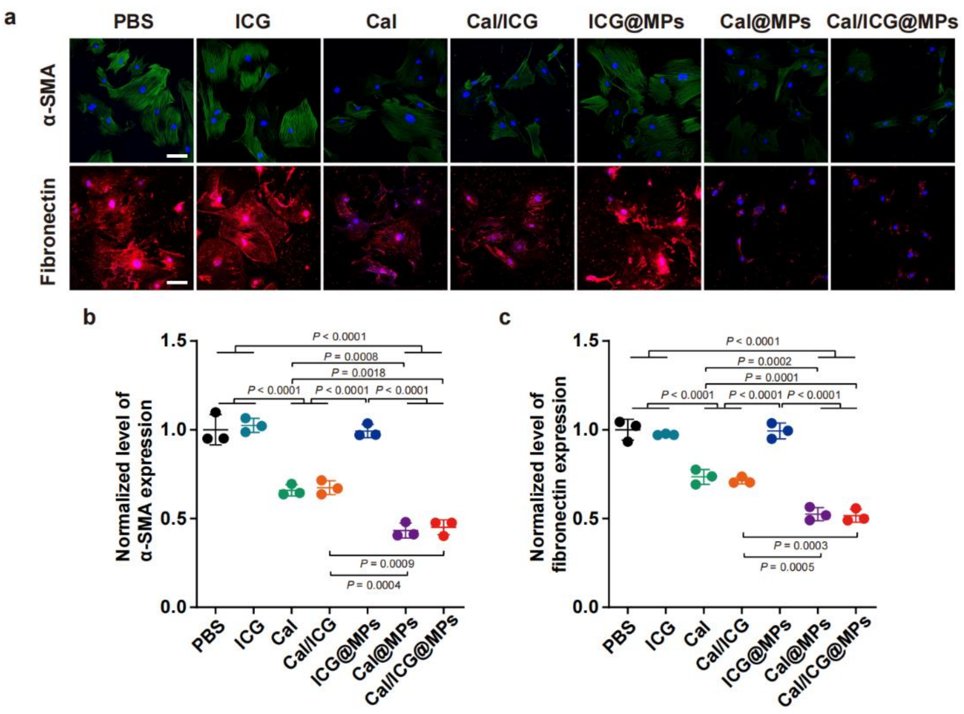

Supplementary Fig. 13 Cal/ICG@MPs-induced myofibroblast regulation and ECM remodeling in vitro.

a Immunofluorescence staining of α-SMA and fibronectin in myofibroblasts after treatment with PBS, ICG, Cal, Cal/ICG, ICG@MPs, Cal@MPs or Cal/ICG@MPs derived from H22 cells at the ICG concentration of 4 μg mL^{-1} and Cal concentration of 60 ng mL^{-1} for 48 h. Images are representative of three biologically independent samples. Scale bars: 100 μm. **b, c** Quantification of the normalized protein expression of α-SMA (**b**) and fibronectin (**c**) indicated in (**a**) using Image J software. Data are presented as means ± s.d. (n = 3 biologically independent samples; one-way ANOVA followed by Tukey's HSD post-hoc test). Source data are provided as a Source Data file.

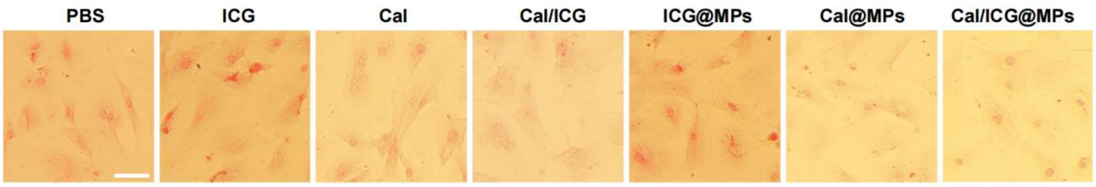

Supplementary Fig. 14 Cal/ICG@MPs-induced collagen deposition in myofibroblasts by Sirius red staining.

Sirius red staining of deposited collagen in myofibroblasts after treatment with PBS, ICG, Cal, Cal/ICG, ICG@MPs, Cal@MPs or Cal/ICG@MPs derived from H22 cells at the ICG concentration of 4 μg mL^{-1} and Cal concentration of 60 ng mL^{-1} for 48 h. Images are representative of three biologically independent samples. Scale bar: 50 μm.

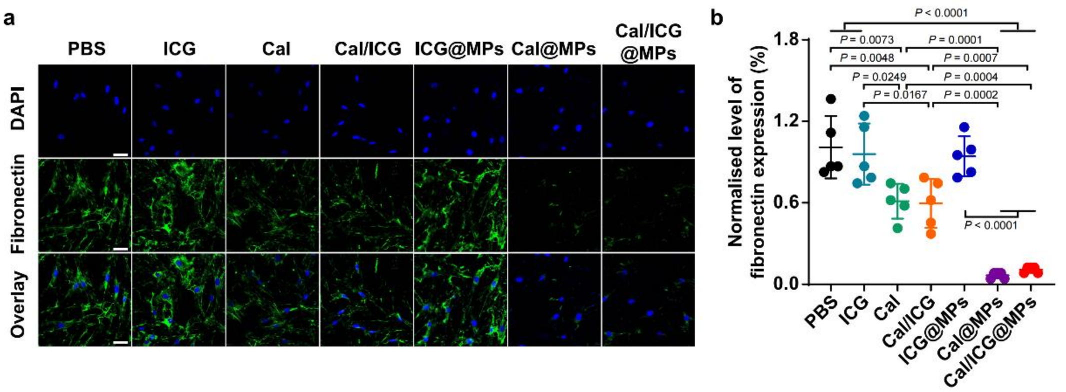

Supplementary Fig. 15 Cal/ICG@MPs-induced regulation in TGF-β-activated human lung fibroblasts.

a Immunofluorescence staining of fibronectin in TGF-β-activated human lung fibroblasts after treatment with PBS, ICG, Cal, Cal/ICG, ICG@MPs, Cal@MPs or Cal/ICG@MPs derived from H22 cells at the ICG concentration of 4 μg mL^{-1} and Cal concentration of 60 ng mL^{-1} for 48 h. Images are representative of five biologically independent samples. Scale bars: 50 μm. **b** Quantification of the normalized protein expression of fibronectin as indicated in (**a**) using Image J software. Data are presented as means ± s.d. (n = 5 biologically independent samples; one-way ANOVA followed by Tukey's HSD post-hoc test). Source data are provided as a Source Data file.

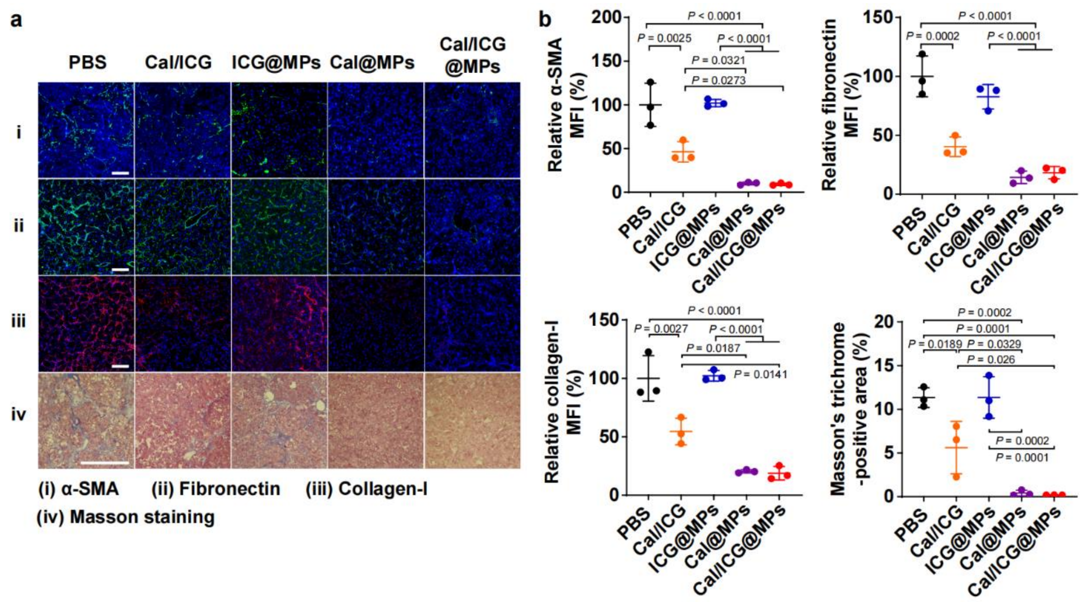

Supplementary Fig. 16 Cal/ICG@MPs-induced ECM remodeling in organotypic slices from liver cancer patient-derived tumor.

a Immunofluorescence staining of α-SMA, fibronectin and collagen-I, and Masson's trichrome staining of collagen in tumor slices from liver cancer patient-derived tumor after treatment with PBS, Cal/ICG, ICG@MPs, Cal@MPs or Cal/ICG@MPs derived from HepG2 cells at the ICG concentration of 4 μg mL^{-1} and Cal concentration of 60 ng mL^{-1} for 48 h. Images are representative of three biologically independent samples. Scale bars: 100 μm. **b** Quantification of α-SMA, fibronectin and collagen-I fluorescence intensity, and positive area of Masson's trichrome staining in tumor slices from liver cancer patient indicated in (**a**) using ImageJ software. Data are presented as means ± s.d. (n = 3 biologically independent samples; one-way ANOVA followed by Tukey's HSD post-hoc test). Source data are provided as a Source Data file.

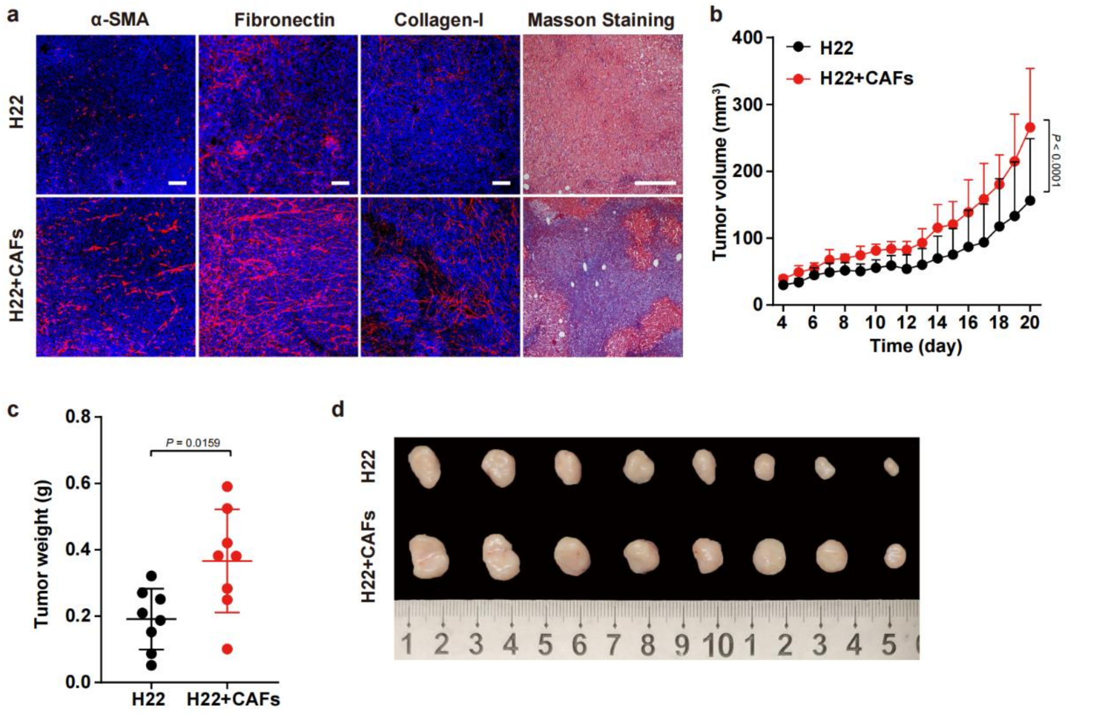

Supplementary Fig. 17 Altered tumor progression and tumor ECM by CAFs in H22 tumor-bearing mice.

a Immunofluorescence staining of α-SMA, fibronectin and collagen-I, and Masson's trichrome staining of collagen in tumor sections of Balb/c mice at 20 days after subcutaneous injection of H22 cells (2×10^6 cells) in the presence or absence of TGF-β-activated skin fibroblasts (1×10^6 cells). Images are representative of three biologically independent mice. Scale bars: 100 μm. **b-d** Average tumor growth curves (**b**), tumor weights (**c**) and tumor images (**d**) in Balb/c mice after subcutaneous injection of H22 cells (2×10^6 cells) in the presence or absence of TGF-β-activated skin fibroblasts (1×10^6 cells). Data are presented as means ± s.d. (n = 8 mice per group; two-way ANOVA followed by Bonferroni's multiple comparisons post-test for **b**, two-tailed unpaired t-test for **c**). Source data are provided as a Source Data file.

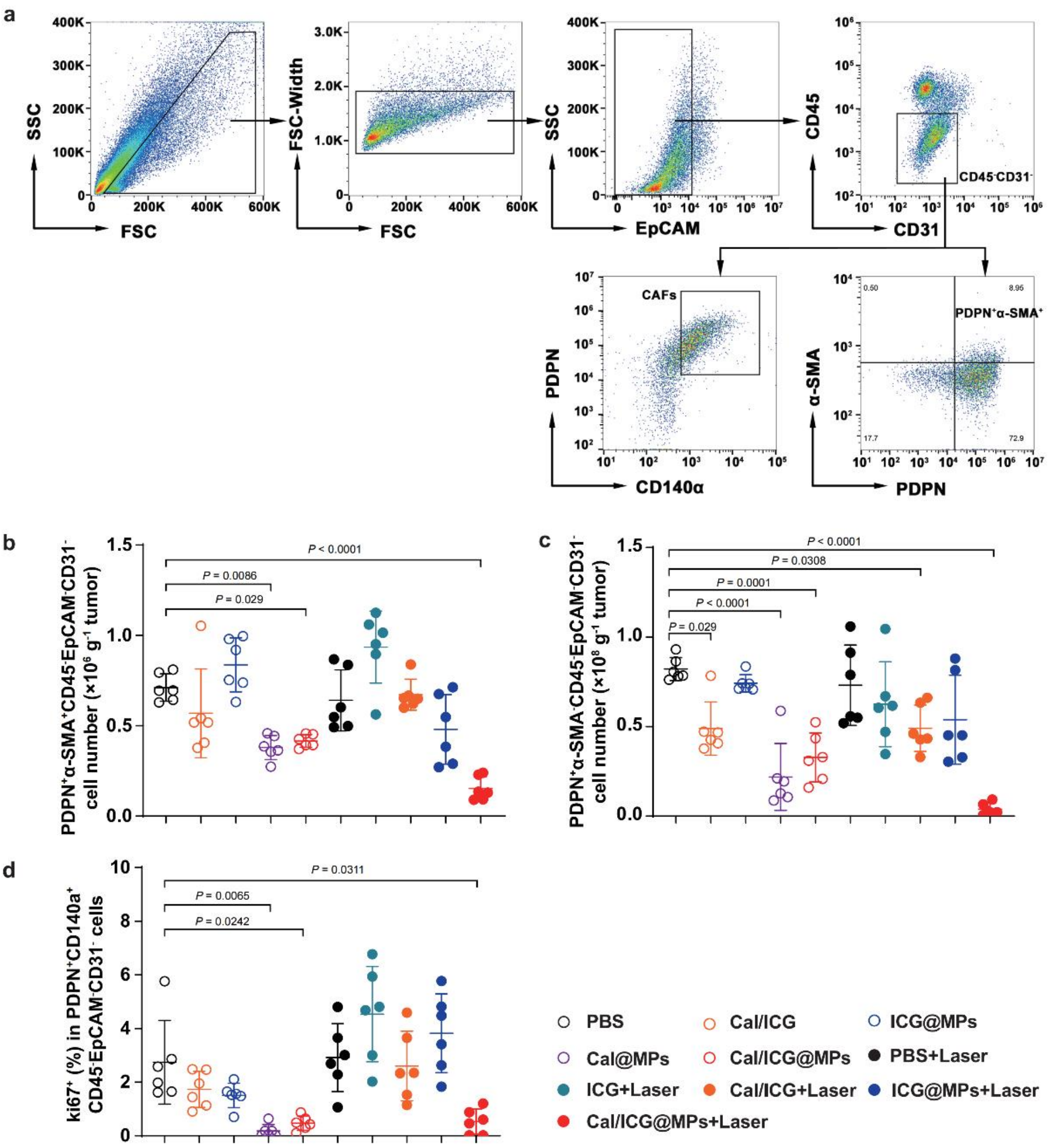

Supplementary Fig. 18 Cal/ICG@MPs-induced CAF regulation in tumor tissues of stroma-rich H22 tumor-bearing mice.

a Gating strategy for identifying CAFs in tumor tissues of tumor-bearing mice presented on Fig. **4c, 5g-i** and Supplementary Fig. **18b-d**. **b-d** Numbers of PDPN$^+$α-SMA$^+$CD45$^-$EpCAM$^-$CD31$^-$ cells (**b**), PDPN$^+$α-SMA$^-$CD45$^-$EpCAM$^-$CD31$^-$ cells (**c**) and percentages of proliferative Ki67-positive CAFs (Ki67$^+$PDPN$^+$CD140α$^+$CD45$^-$EpCAM$^-$CD31$^-$, **d**) in tumor tissues of stroma-rich H22 tumor-bearing mice after intravenous injection of PBS, ICG, Cal/ICG, ICG@MPs, Cal@MPs or Cal/ICG@MPs

derived from H22 cells at the ICG dosage of 8 mg kg^{-1} and Cal dosage of 120 μg kg^{-1} twice every two days, followed with or without 808 nm laser irradiation (1.5 W cm^{-2}, 10 min) at 2 h after the last injection. Data are presented as means ± s.d. (n = 6 mice per group; one-way ANOVA followed by Tukey's HSD post-hoc test). Source data are provided as a Source Data file.

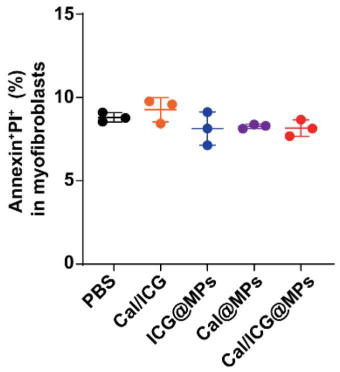

Supplementary Fig. 19 Effects of Cal/ICG@MPs on the apoptosis induction in myofibroblasts.

Annexin-V/PI assay of myofibroblasts after treatment with PBS, Cal/ICG, ICG@MPs, Cal@MPs or Cal/ICG@MPs derived from H22 cells at the ICG concentration of 4 μg mL^{-1} and Cal concentration of 60 ng mL^{-1} for 48 h. Data are presented as means ± s.d. (n = 3 biologically independent samples). Source data are provided as a Source Data file.

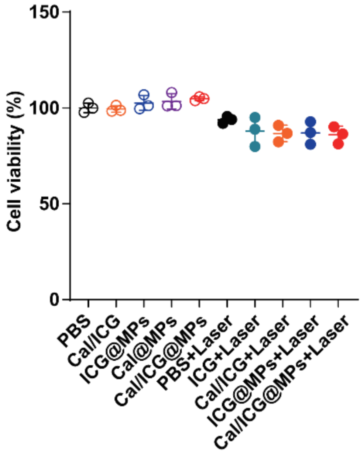

Supplementary Fig. 20 Cal/ICG@MPs-triggered cytotoxicity against myofibroblasts upon 808 nm laser irradiation.

Cell viability of myofibroblasts after treatment with PBS, ICG, Cal/ICG, ICG@MPs, Cal@MPs or Cal/ICG@MPs derived from H22 cells at the ICG concentration of 4 μg mL^{-1} for 4 h in the presence or absence of 808 nm laser irradiation (1 W cm^{-2}, 5 min) by MTT assay. Data are presented as means ± s.d. (n = 3 biologically independent samples). Source data are provided as a Source Data file.

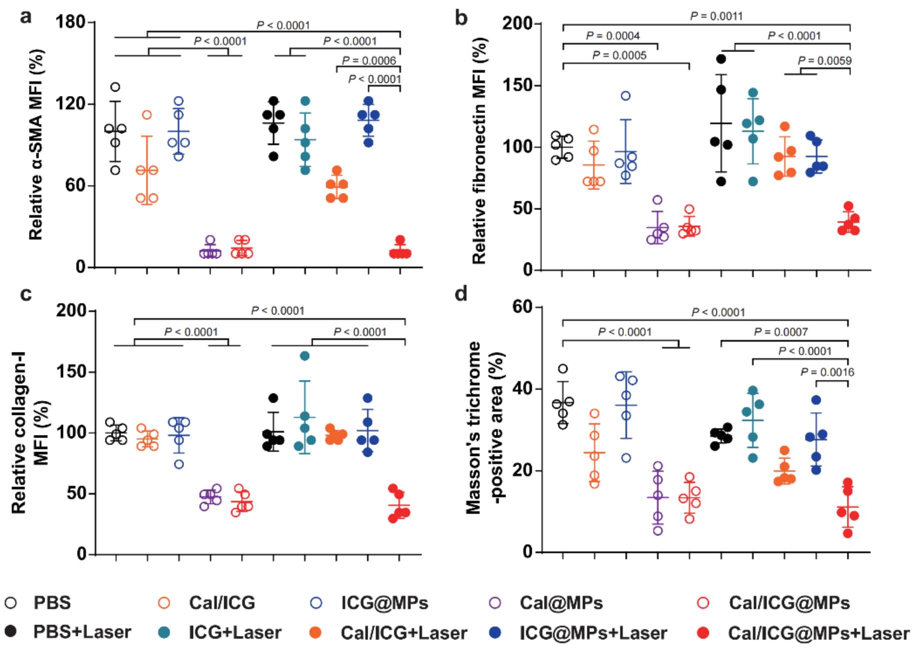

Supplementary Fig. 21 Cal/ICG@MPs-induced ECM remodeling in tumor tissues of stroma-rich H22 tumors.

a-d Quantification of α-SMA(**a**), fibronectin(**b**) and collagen-I (**c**) fluorescence intensity, and positive area of Masson's trichrome staining (**d**) in tumor tissues of stroma-rich H22 tumor-bearing mice as indicated in Fig. **4d** using ImageJ software. Data are presented as means ± s.d. (n = 5 fields in total from 3 mice; one-way ANOVA followed by Tukey's HSD post-hoc test). Source data are provided as a Source Data file.

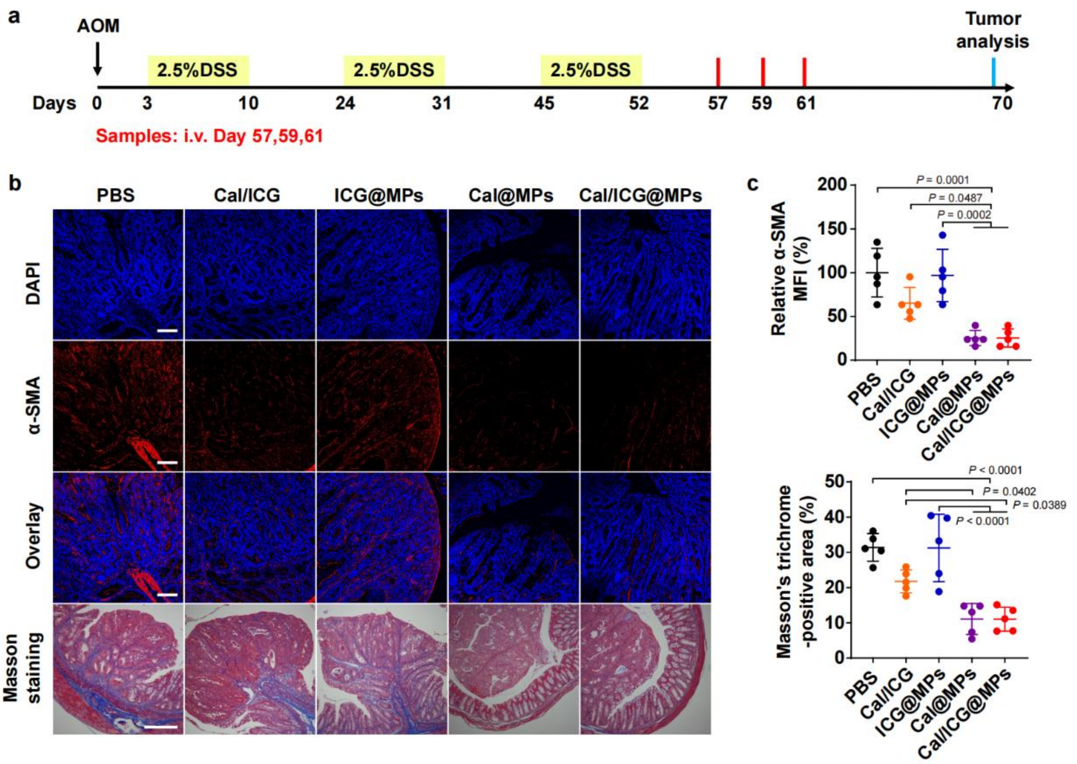

Supplementary Fig. 22 Cal/ICG@MPs-induced ECM remodeling in the tumors of AOM/DSS-induced CAC mouse model.

a Schematic schedule for the ECM remodeling experiment in AOM/DSS-induced CAC mice after intravenous injection of PBS, Cal/ICG, ICG@MPs, Cal@MPs or Cal/ICG@MPs derived from MC38 cells at the ICG dosage of 8 mg kg^{-1} and Cal dosage of 120 μg kg^{-1} every two days for three times. **b** Immunofluorescence staining of α-SMA and Masson's trichrome staining of collagen in the colon tumor sections of AOM/DSS-induced CAC mice after treatment indicated in (**a**). Images are representative of three biologically independent mice. Scale bars: 100 μm. **c** Quantification of α-SMA fluorescence intensity and positive area of Masson's trichrome staining in the colon tumor sections of AOM/DSS-induced CAC mouse model indicated in (**b**) using ImageJ software. Data are presented as means ± s.d. (n = 5 fields in total from 3 mice; one-way ANOVA followed by Tukey's HSD post-hoc test). Source data are provided as a Source Data file.

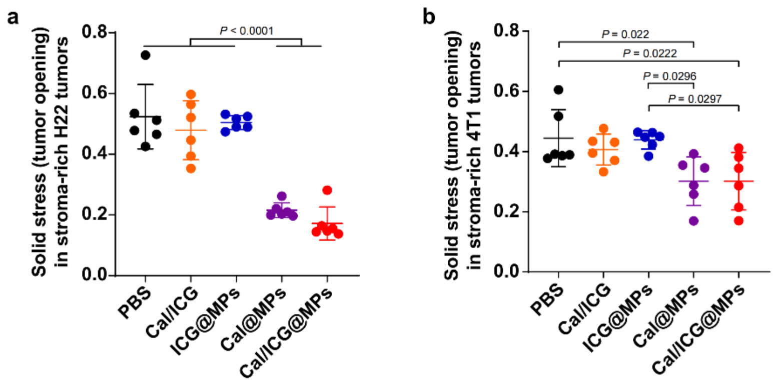

Supplementary Fig. 23 Cal/ICG@MPs-induced decrease in solid stress of tumors. **a, b** Solid stress of tumors of stroma-rich H22 tumor-bearing mice (**a**) and 4T1 tumor-bearing mice (**b**) at 14 days after intravenous injection of PBS, Cal/ICG, ICG@MPs, Cal@MPs or Cal/ICG@MPs derived from H22 and 4T1 cells, at the ICG dosage of 8 mg kg^{-1} and Cal dosage of 120 μg kg^{-1} twice every two days, respectively, which was assessed using an ex vivo technique involving the measurement of the extent of tumor tissue relaxation (tumor opening relative to tumor diameter) following a stress-releasing incision, with larger openings indicating higher stress. Data are presented as means ± s.d. (n = 6 mice per group; one-way ANOVA followed by Tukey's HSD post-hoc test). Source data are provided as a Source Data file.

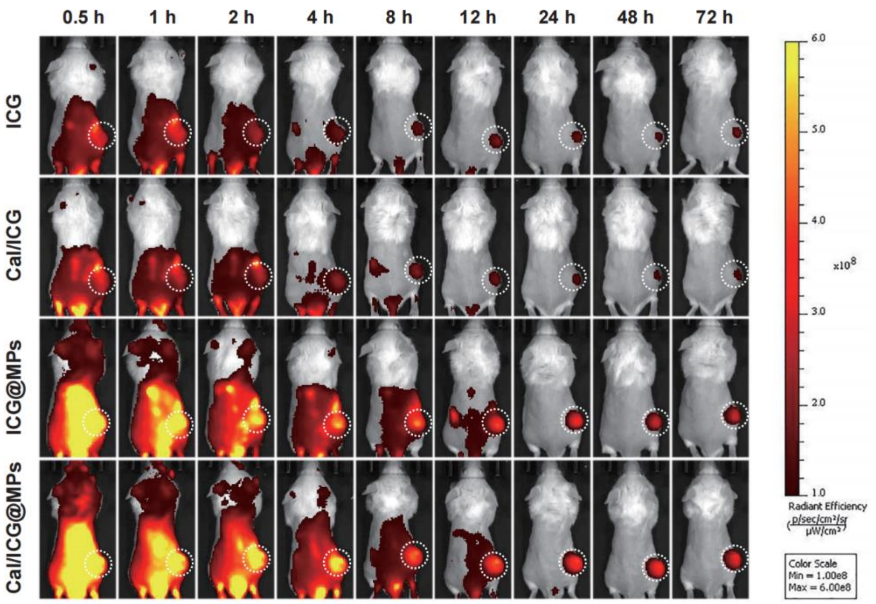

Supplementary Fig. 24 In vivo NIRF imaging of H22 tumor-bearing mice.

In vivo NIRF images of stroma-rich H22 tumor-bearing mice (constructed by co-injection of H22 cells and TGF-β-activated skin fibroblasts) at different time intervals after intravenous injection of ICG, Cal/ICG, ICG@MPs or Cal/ICG@MPs derived from H22 cells at the ICG dosage of 5 mg kg-1 and Cal dosage of 75 μg kg^{-1} twice every two days. Images are representative of three biologically independent mice.

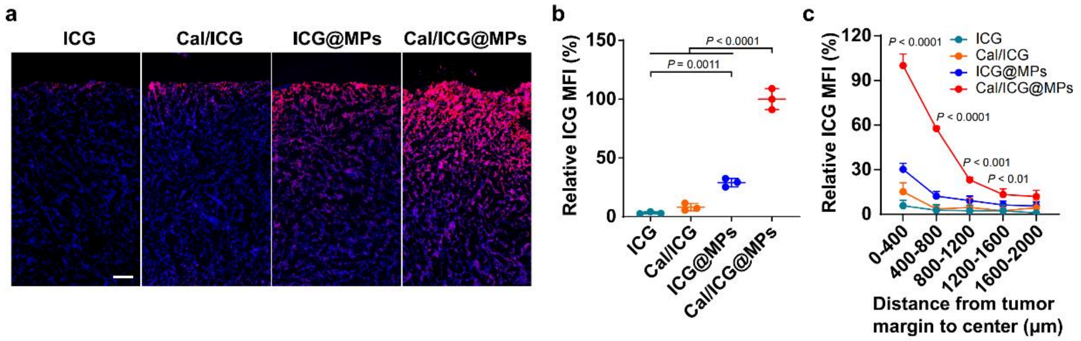

Supplementary Fig. 25 Cal/ICG@MPs-enhanced tumor accumulation and penetration in organotypic slices from liver cancer patient-derived tumor.

a ICG fluorescence images in organotypic liver cancer patient-derived tumor slices after treatment with ICG, Cal/ICG, ICG@MPs or Cal/ICG@MPs derived from HepG2 cells at the ICG concentration of 4 μg mL^{-1} and Cal concentration of 60 ng mL^{-1} for 48 h. Images are representative of three biologically independent samples. Scale bar: 200 μm. **b, c** Quantification of ICG fluorescence intensity (**b**) and ICG distribution profiles from tumor margin to center (**c**) in organotypic tumor slices as indicated in (**a**) using ImageJ software. Data are presented as means ± s.d. (n = 3 biologically independent samples; one-way ANOVA followed by Tukey's HSD post-hoc test for **b**, two-way ANOVA followed by Tukey's multiple comparisons post-test for **c**). Source data are provided as a Source Data file.

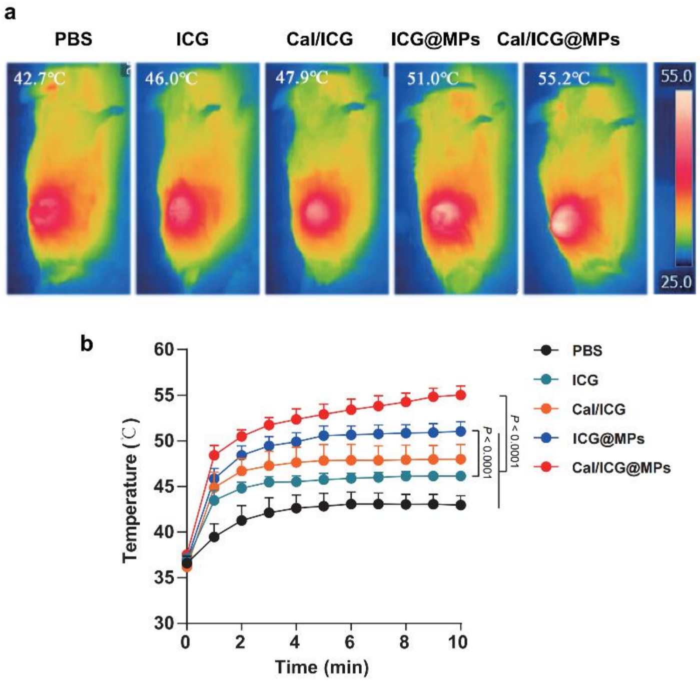

Supplementary Fig. 26 Photothermal effects of Cal/ICG@MPs in stroma-rich H22 tumor-bearing mice.

a, b IR thermal images (**a**) and temperature curves (**b**) of stroma-rich H22 tumor-bearing mice (constructed by co-injection of H22 cells and HSCs) after intravenous injection of PBS, ICG, Cal/ICG, ICG@MPs or Cal/ICG@MPs derived from H22 cells at the ICG dosage of 8 mg kg^{-1} and Cal dosage of 120 μg kg^{-1} twice every two days, followed with 808 nm laser irradiation (1.5 W cm^{-2}, 10 min) at 2 h after the last injection. Data are presented as means ± s.d. (n = 6 mice per group; two-way ANOVA followed by Tukey's multiple comparisons post-test). Source data are provided as a Source Data file.

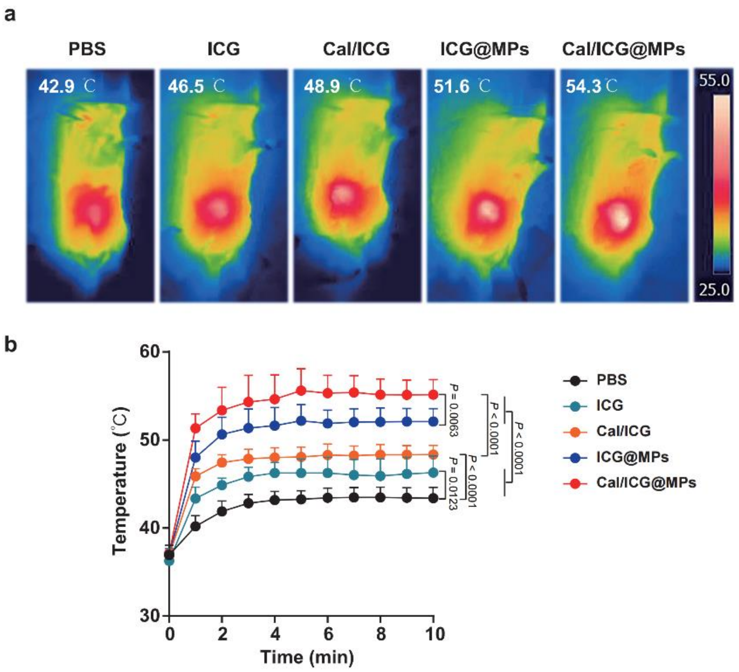

Supplementary Fig. 27 Photothermal effects of Cal/ICG@MPs in stroma-rich 4T1 tumor-bearing mice.

a, b IR thermal images (**a**) and temperature curves (**b**) of stroma-rich 4T1 tumor-bearing mice (constructed by co-injection of 4T1 cells and TGF-β-activated skin fibroblasts) after intravenous injection of PBS, ICG, Cal/ICG, ICG@MPs or Cal/ICG@MPs derived from 4T1 cells at the ICG dosage of 8 mg kg^{-1} and Cal dosage of 120 μg kg^{-1} twice every two days, followed with 808 nm laser irradiation (1.5 W cm^{-2}, 10 min) at 2 h after the last injection. Data are presented as means ± s.d. (n = 6 mice per group; two-way ANOVA followed by Tukey's multiple comparisons post-test). Source data are provided as a Source Data file.

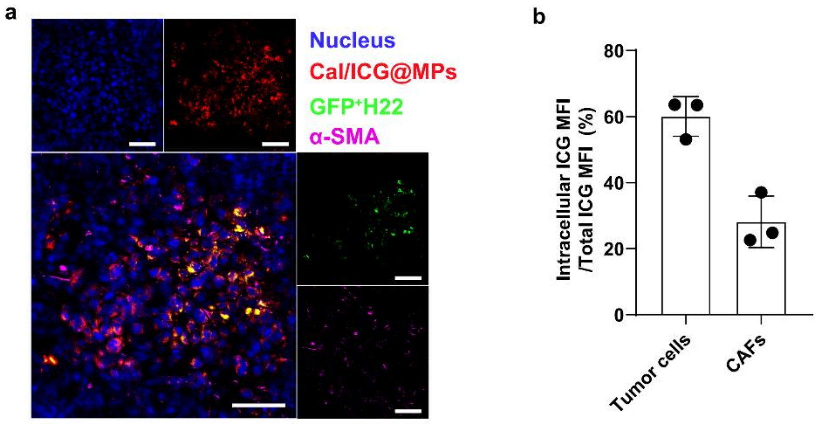

Supplementary Fig. 28 Distribution of Cal/ICG@MPs in tumor cells and CAFs of stroma-rich H22 tumor-bearing mice.

a Representative images of Cal/ICG@MPs (red) distribution in tumors of stroma-rich H22 tumor-bearing mice (constructed by co-injection of GFP-overexpressed H22 cells and TGF-β-activated skin fibroblasts) at 48 h after intravenous injection of Cal/ICG@MPs derived from H22 cells at the ICG dosage of 8 mg kg^{-1} and Cal dosage of 120 μg kg^{-1}. CAFs were labeled with anti-αSMA antibody (purple) and tumor cells were GFP^{+} cells (green). Images are representative of two biologically independent mice. Scale bars: 40 μm. **b** Quantification of Cal/ICG@MPs in tumor cells and CAFs indicated in (**a**) using ImageJ software. Data are presented as means ± s.d. (n = 3 fields in total from 2 mice). Source data are provided as a Source Data file.

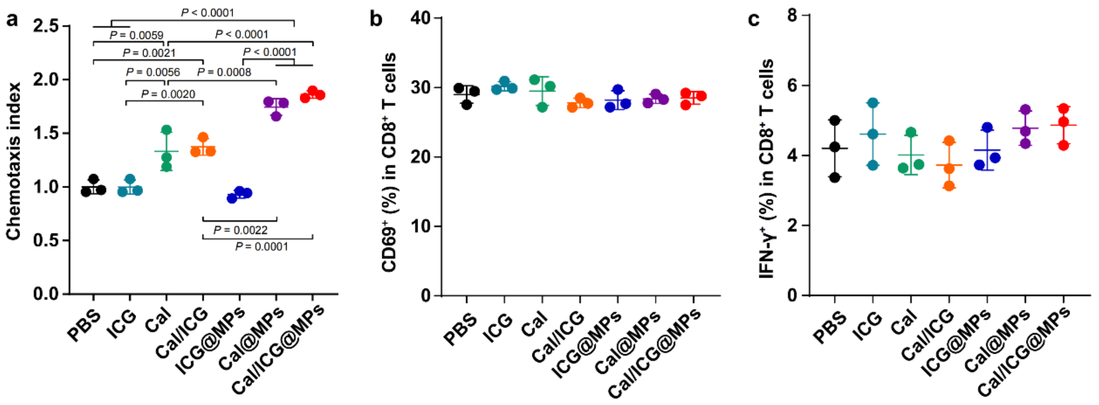

Supplementary Fig. 29 Chemotaxis activity of CD8^{+} T cells by Cal/ICG@MPs-regulated CAFs.

a Chemotaxis index of CD3^{+}CD8^{+} T cells at 8 h after CD3^{+} T cells isolated from BALB/c mice were seeded in the top chambers and the supernatants of CAFs pretreated with PBS, ICG, Cal, Cal/ICG, ICG@MPs, Cal@MPs or Cal/ICG@MPs derived from H22 cells at the ICG concentration of 4 μg mL^{-1} and Cal concentration of 60 ng mL^{-1} for 48 h were added into the bottom chambers by flow cytometry. Data are presented as means ± s.d. (n = 3 biologically independent samples; one-way ANOVA followed by Tukey's HSD post-hoc test). **b, c** Percentages of CD69^{+} (**b**) and IFN-γ^{+} (**c**) cells in CD3^{+}CD8^{+} T cells after treatment indicated in (**a**). Data are presented as means ± s.d. (n = 3 biologically independent samples). Source data are provided as a Source Data file.

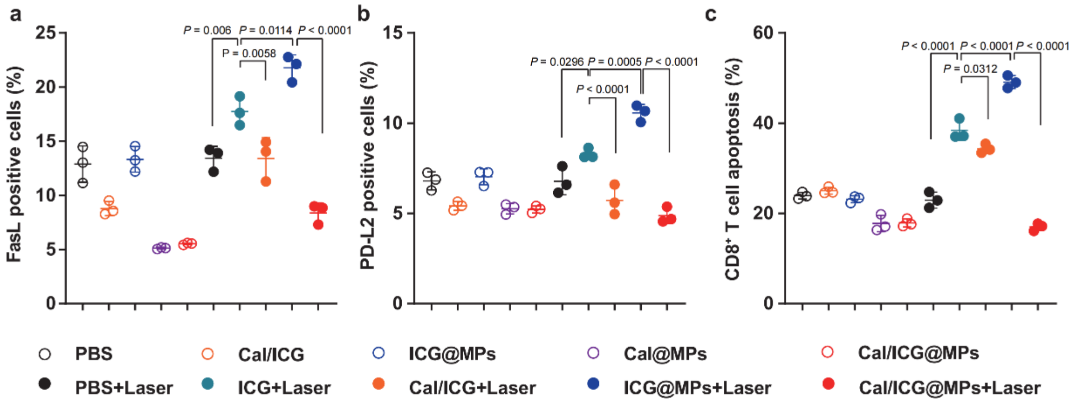

Supplementary Fig. 30 Attenuated myofibroblast-induced antigen-mediated AICD effects of CD8$^+$ T cells by Cal/ICG@MPs.

a, b FasL (**a**) and PD-L2 (**b**) expression in myofibroblasts after myofibroblasts pretreated with PBS, ICG, Cal/ICG, ICG@MPs, Cal@MPs or Cal/ICG@MPs derived from 4T1 cells at the ICG concentration of 4 μg mL^{-1} and Cal concentration of 60 ng mL^{-1} for 48 h were co-cultured with the cell supernatants from the corresponding same formulations with or without 808 nm laser irradiation-treated 4T1 cells for 24 h and then CD8$^+$ T cells for another 48 h as indicated in Fig. **5a**. Data are presented as means ± s.d. (n = 3 biologically independent samples; one-way ANOVA followed by Tukey's HSD post-hoc test). **c** Apoptosis ratio of CD8$^+$ T cells after treatment indicated in (**a**). Data are presented as means ± s.d. (n = 3 biologically independent samples; one-way ANOVA followed by Tukey's HSD post-hoc test). Source data are provided as a Source Data file.

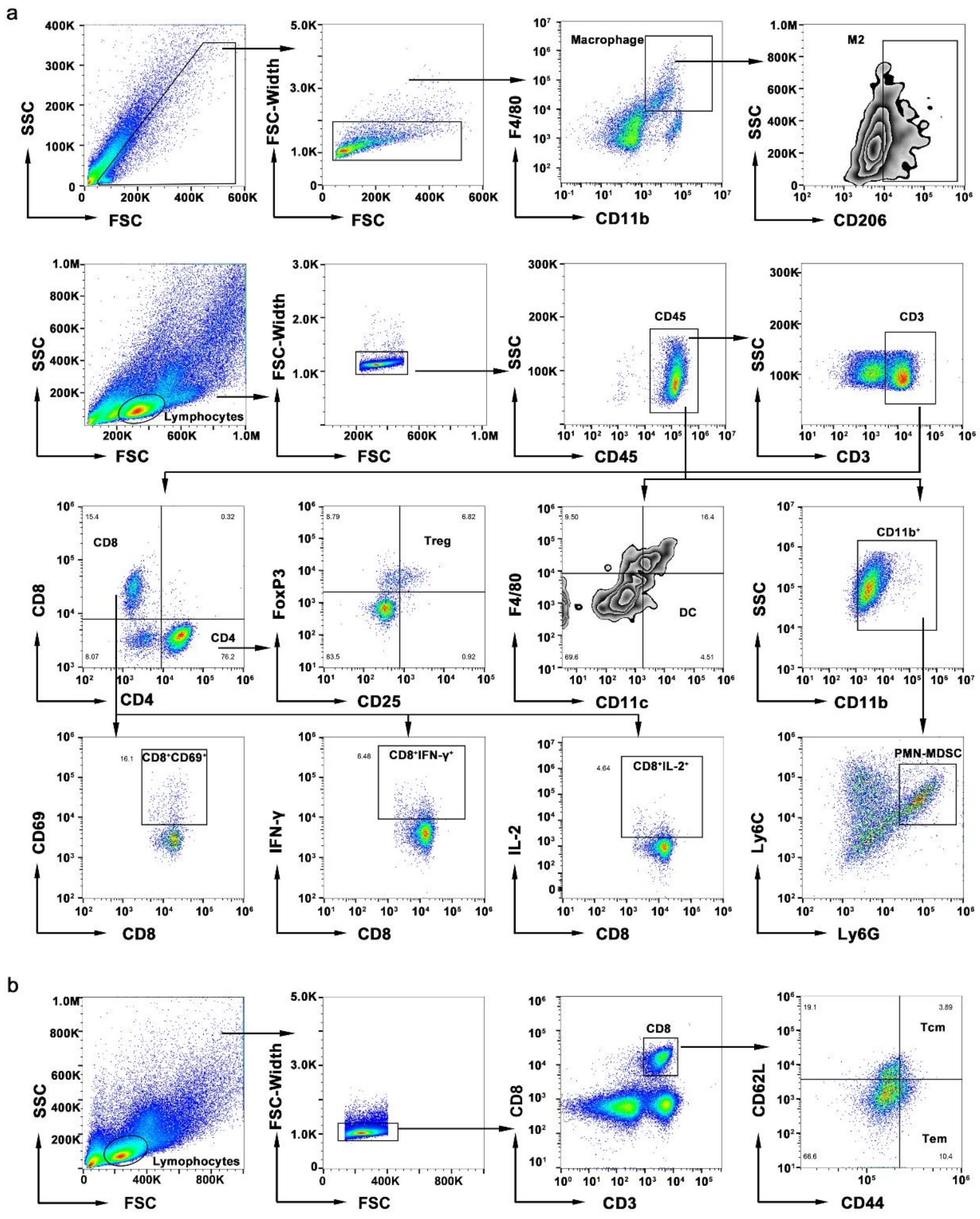

Supplementary Figure 31. Gating Strategy for identifying major immune cell populations in tumor tissues or spleens of tumor-bearing mice.

a Gating strategy for identifying M2-like TAMs, $CD8^+$ T cells, $CD8^+CD69^+$ T cells, $CD8^+$IFN-γ^+ T cells, $CD8^+$IL-2^+ T cells, Tregs, DCs and PMN-MDSCs in tumor tissues of tumor-bearing mice presented on Fig **5j, 5k, 6b-e** and Supplementary Fig **32a-k**. **b** Gating strategy for identifying Tcm and Tem in spleens of tumor-bearing mice presented on Fig **7e,7f**.

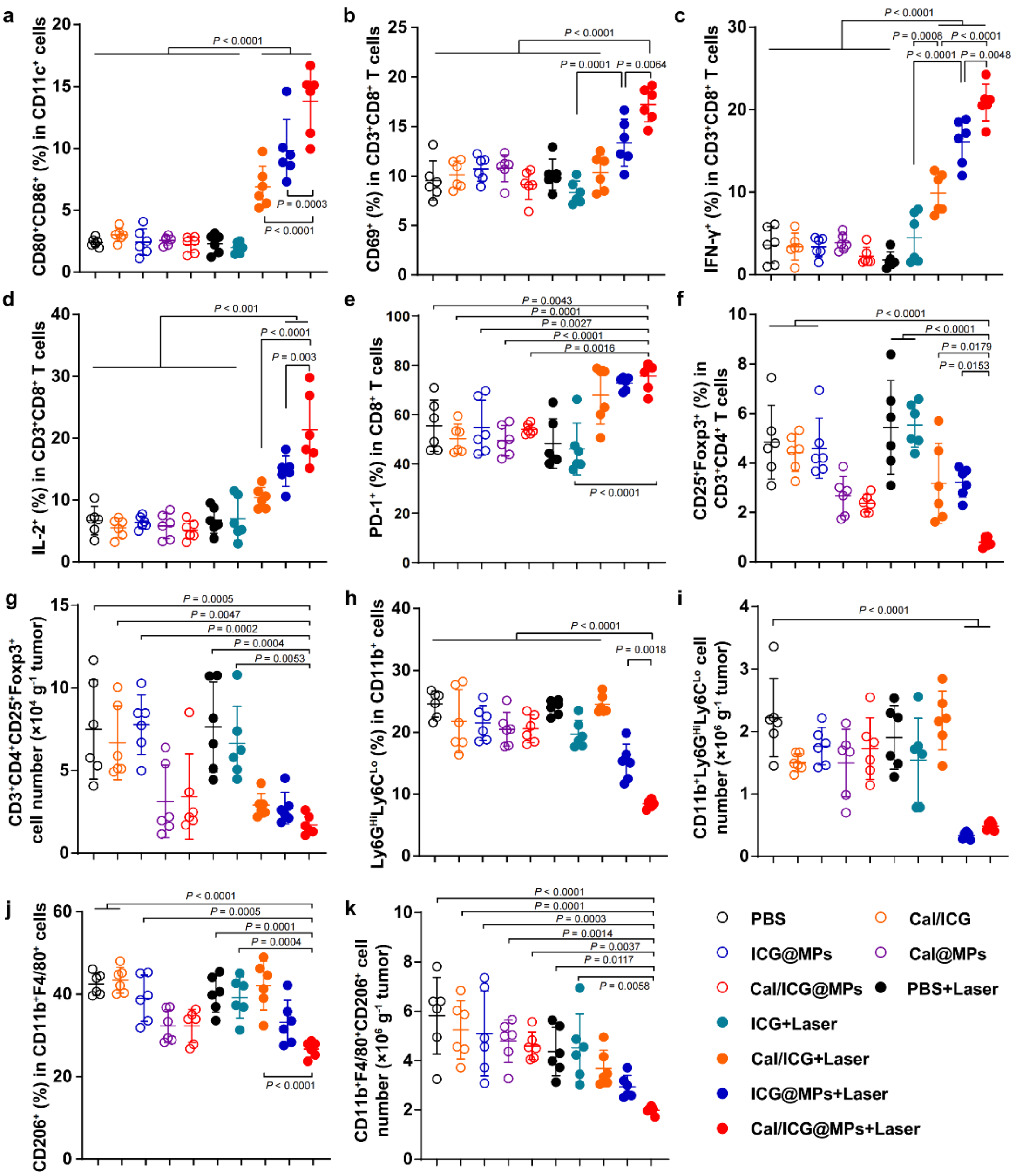

Supplementary Fig. 32 Cal/ICG@MPs-induced improved immune microenvironment in tumors of stroma-rich H22 tumor-bearing mice upon 808 nm laser irradiation.

a-k Percentages of DC maturation (**a**), $CD8^+CD69^+$ T cells (**b**), $CD8^+IFN\text{-}\gamma^+$ T cells (**c**), $CD8^+IL\text{-}2^+$ T cells (**d**), $CD8^+PD\text{-}1^+$ T cells (**e**), Tregs (**f**), PMN-MDSCs (**h**), M2-like TAMs (**j**) and numbers of Tregs (**g**), PMN-MDSCs (**i**), M2-like TAMs (**k**) in tumor tissues of stroma-rich H22 tumor-bearing mice (constructed by co-injection of H22

cells and TGF-β-activated skin fibroblasts) after intravenous injection of PBS, ICG, Cal/ICG, ICG@MPs, Cal@MPs or Cal/ICG@MPs derived from H22 cells at the ICG dosage of 8 mg kg^{-1} and Cal dosage of 120 μg kg^{-1} twice every two days, followed with or without 808 nm laser irradiation (1.5 W cm^{-2}, 10 min) at 2 h after the last injection. Data are presented as means ± s.d. (n = 6 mice per group; one-way ANOVA followed by Tukey's HSD post-hoc test). Source data are provided as a Source Data file.

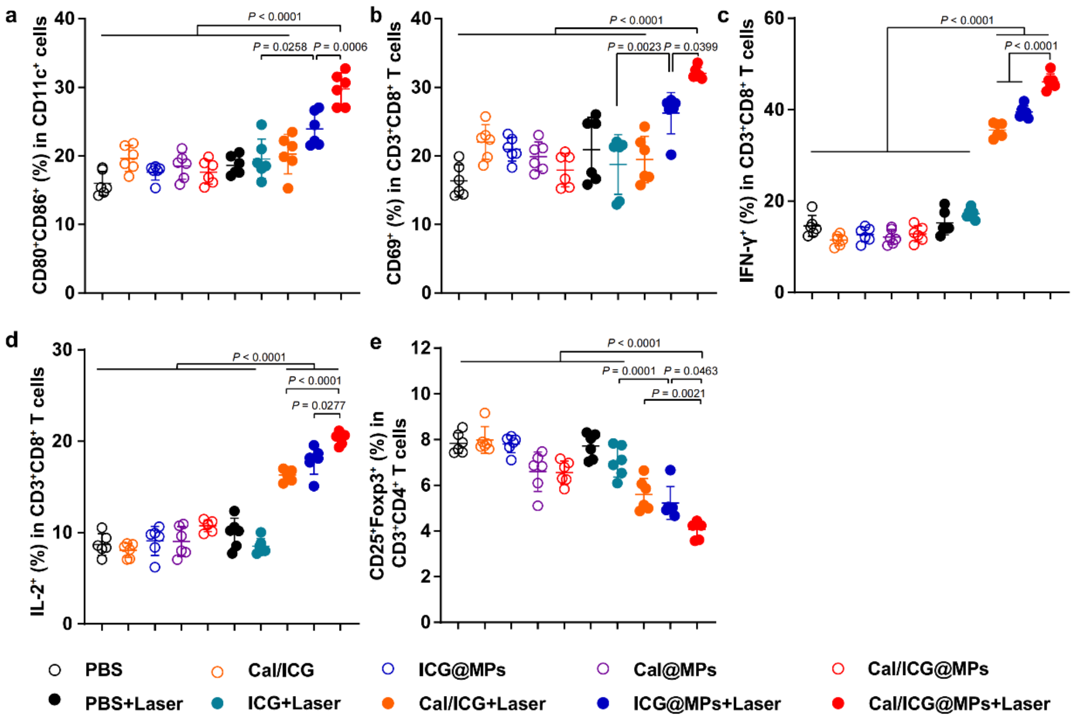

Supplementary Fig. 33 Cal/ICG@MPs-induced improved immune responses in tumor draining LNs of stroma-rich H22 tumor-bearing mice upon 808 nm laser irradiation.

a-e Percentages of DC maturation (**a**), $CD8^+CD69^+$ T cells (**b**), $CD8^+IFN\text{-}\gamma^+$ T cells (**c**), $CD8^+IL\text{-}2^+$ T cells (**d**) and Tregs (**e**) in tumor draining LNs of stroma-rich H22 tumor-bearing mice (constructed by co-injection of H22 cells and TGF-β-activated skin fibroblasts) after intravenous injection of PBS, ICG, Cal/ICG, ICG@MPs, Cal@MPs or Cal/ICG@MPs derived from H22 cells at the ICG dosage of 8 mg kg^{-1} and Cal dosage of 120 μg kg^{-1} twice every two days, followed with or without 808 nm laser irradiation (1.5 W cm^{-2}, 10 min) at 2 h after the last injection. Data are presented as means ± s.d. (n = 6 mice per group; one-way ANOVA followed by Tukey's HSD post-hoc test). Source data are provided as a Source Data file.

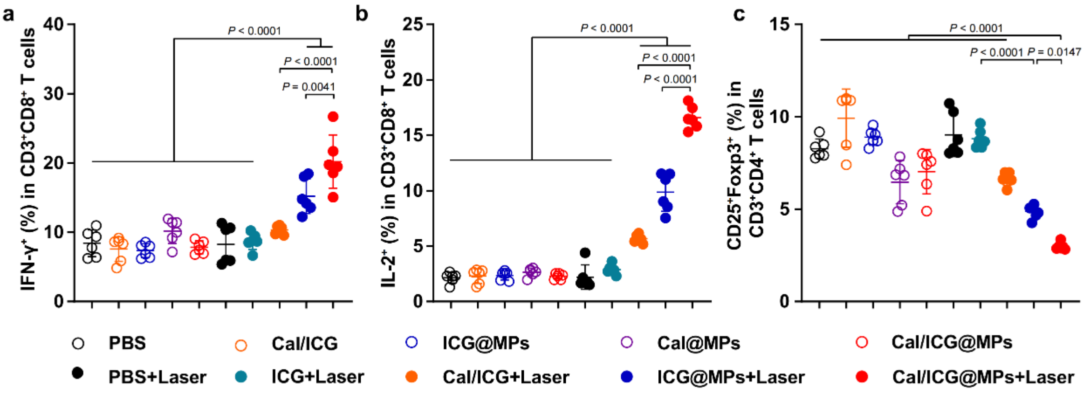

Supplementary Fig. 34 Cal/ICG@MPs-induced improved immune responses in spleens of stroma-rich H22 tumor-bearing mice upon 808 nm laser irradiation. **a-c** Percentages of $CD8^+IFN\text{-}\gamma^+$ T cells (**a**), $CD8^+IL\text{-}2^+$ T cells (**b**) and Tregs (**c**) in spleens of stroma-rich H22 tumor-bearing mice (constructed by co-injection of H22 cells and TGF-β-activated skin fibroblasts) after intravenous injection of PBS, ICG, Cal/ICG, ICG@MPs, Cal@MPs or Cal/ICG@MPs derived from H22 cells at the ICG dosage of 8 mg kg^{-1} and Cal dosage of 120 μg kg^{-1} twice every two days, followed with or without 808 nm laser irradiation (1.5 W cm^{-2}, 10 min) at 2 h after the last injection. Data are presented as means ± s.d (n = 6 mice per group; one-way ANOVA followed by Tukey's HSD post-hoc test). Source data are provided as a Source Data file.

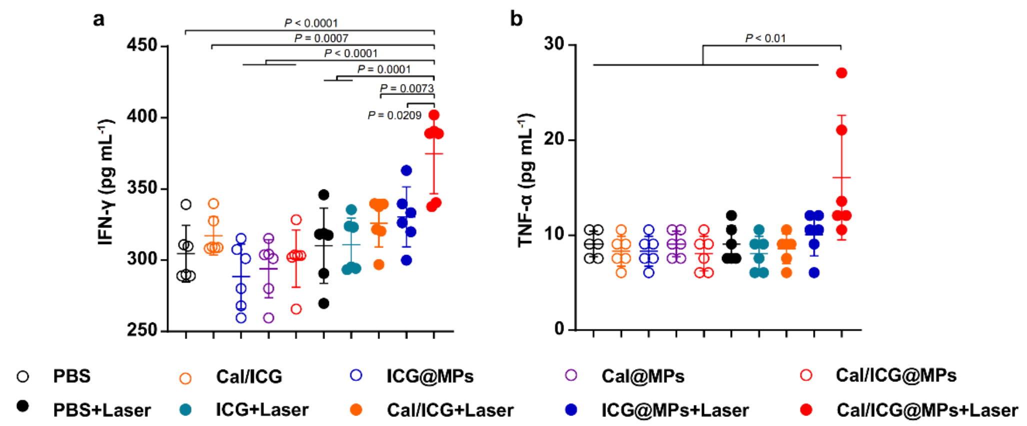

Supplementary Fig. 35 Cal/ICG@MPs-induced Th1 cytokine production in stroma-rich H22 tumor-bearing mice upon 808 nm laser irradiation.

a, b IFN-γ (**a**) and TNF-α contents (**b**) in serum of stroma-rich H22 tumor-bearing mice (constructed by co-injection of H22 cells and TGF-β-activated skin fibroblasts) after intravenous injection of PBS, ICG, Cal/ICG, ICG@MPs, Cal@MPs or Cal/ICG@MPs derived from H22 cells at the ICG dosage of 8 mg kg^{-1} and Cal dosage of 120 μg kg^{-1} twice every two days, followed with or without 808 nm laser irradiation (1.5 W cm^{-2}, 10 min) at 2 h after the last injection. Data are presented as means ± s.d. (n = 6 mice per group; one-way ANOVA followed by Tukey's HSD post-hoc test). Source data and exact *P* values for **b** are provided as a Source Data file.

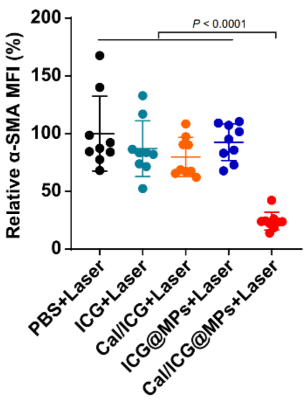

Supplementary Fig. 36 Quantification of α-SMA in tumor tissues of stroma-rich H22 tumor-bearing mice.

Quantification of α-SMA in tumor tissues of stroma-rich H22 tumor-bearing mice (constructed by co-injection of H22 cells and TGF-β-activated skin fibroblasts) after treatment indicated in Fig. **6f** using ImageJ software. Data are presented as means ± s.d. (n = 9 fields in total from 3 mice; one-way ANOVA followed by Tukey's HSD post-hoc test). Source data are provided as a Source Data file.

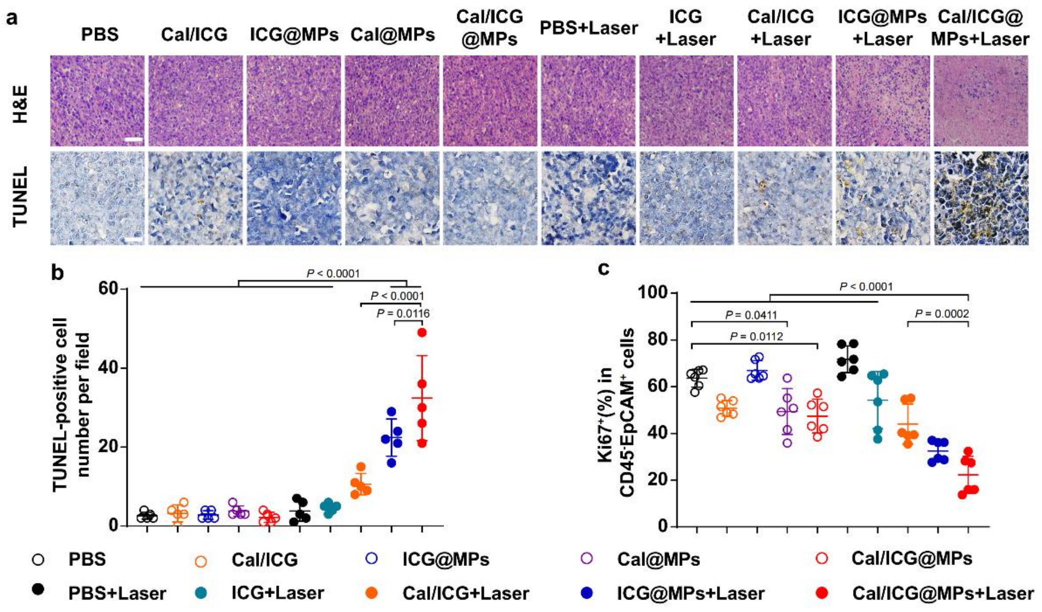

Supplementary Fig. 37 Anticancer activity of Cal/ICG@MPs in stroma-rich H22 tumor-bearing upon 808 nm laser irradiation by histological observation and Ki67 analysis.

a Representative H&E and TUNEL staining images of tumor sections in stroma-rich H22 tumor-bearing mice (constructed by co-injection of H22 cells and TGF-β-activated skin fibroblasts) after intravenous injection of PBS, ICG, Cal/ICG, ICG@MPs, Cal@MPs or Cal/ICG@MPs derived from H22 cells at the ICG dosage of 8 mg kg^{-1} and Cal dosage of 120 μg kg^{-1} twice every two days, followed with or without 808 nm laser irradiation (1.5 W cm^{-2}, 10 min) at 2 h after the last injection. Images are representative of three biologically independent mice. Scale bars: 20 μm. **b** Quantification of TUNEL-positive cells in tumor tissues as indicated in (**a**) using ImageJ software. Data are presented as means ± s.d. (n = 5 fields in total from 3 mice; one-way ANOVA followed by Tukey's HSD post-hoc test). **c** Percentage of Ki67-positive proliferative tumor cells ($CD45^-EpCAM^+Ki67^+$) in tumor tissues of stroma-rich H22 tumor-bearing mice after treatment indicated in (**a)** by flow cytometry. Data are presented as means ± s.d. (n = 6 mice per group; one-way ANOVA followed by Tukey's HSD post-hoc test). Source data are provided as a Source Data file.

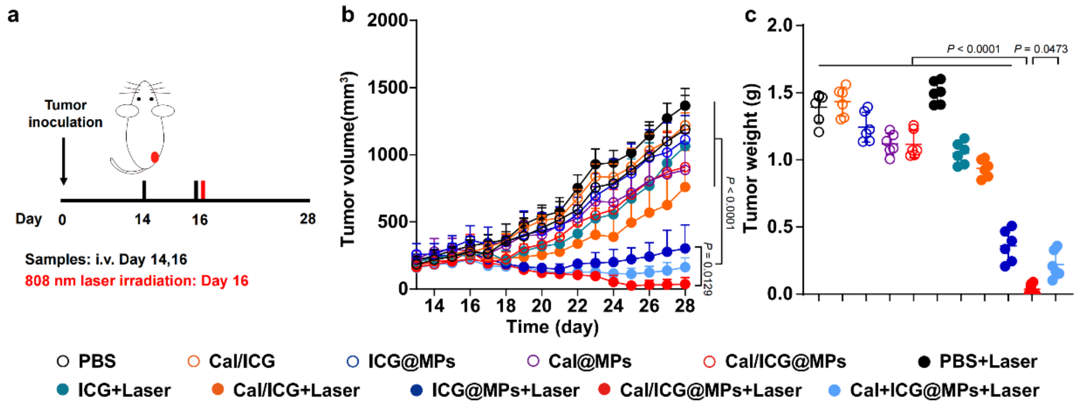

Supplementary Fig. 38 Anticancer activity of Cal/ICG@MPs in stroma-rich H22 tumor-bearing mice upon 808 nm laser irradiation.

a Schematic schedule for the antitumor experiment in stroma-rich H22 tumor-bearing mice (constructed by co-injection of H22 cells and HSCs) after intravenous injection of PBS, ICG, Cal/ICG, ICG@MPs, Cal@MPs, combination of ICG@MPs and Cal (Cal+ICG@MPs) or Cal/ICG@MPs derived from H22 cells at the ICG dosage of 8 mg kg^{-1} and Cal dosage of 120 μg kg^{-1}, followed with or without 808 nm laser irradiation (1.5 W cm^{-2}, 10 min) at 2 h after the last injection. **b, c** Average tumor growth curves (**b**) and tumor weights (**c**) of stroma-rich H22 tumor-bearing mice after treatment indicated in (**a**). Data are presented as means ± s.d. (n = 6 mice per group; two-way ANOVA followed by Tukey's multiple comparisons post-test for **b**, one-way ANOVA followed by Tukey's HSD post-hoc test for **c**). Source data are provided as a Source Data file.

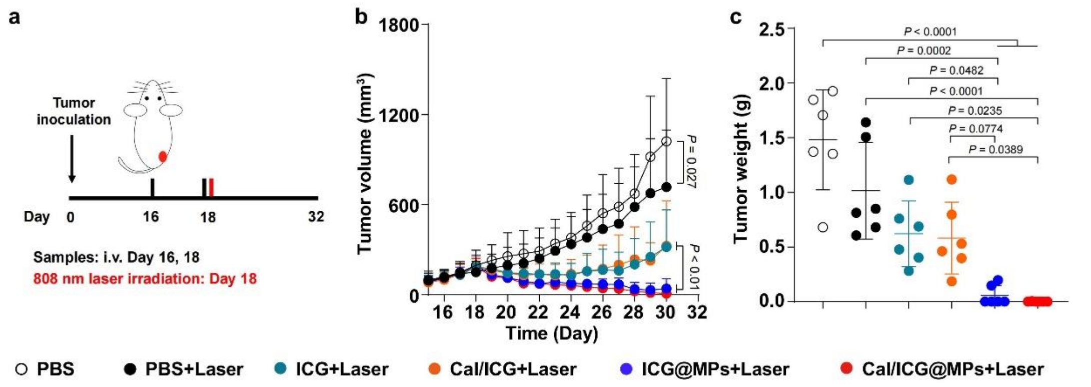

Supplementary Fig. 39 Anticancer activity of Cal/ICG@MPs in stroma-poor H22 tumor bearing mice upon 808 nm laser irradiation.

a Schematic schedule for the antitumor experiment in stroma-poor H22 tumor-bearing mice (constructed by injection of only H22 cells) after intravenous injection of PBS, ICG, Cal/ICG, ICG@MPs, Cal@MPs or Cal/ICG@MPs derived from H22 cells at the ICG dosage of 8 mg kg^{-1} and Cal dosage of 120 μg kg^{-1}, followed with or without 808 nm laser irradiation (1.5 W cm^{-2}, 10 min) at 2 h after the last injection. **b, c** Average tumor growth curves (**b**) and tumor weights (**c**) of stroma-poor H22 tumor-bearing mice after treatment indicated in (**a**). Data are presented as means ± s.d. (n = 6 mice per group; two-way ANOVA followed by Tukey's multiple comparisons post-test for **b**, one-way ANOVA followed by Tukey's HSD post-hoc test for **c**). Source data are provided as a Source Data file.

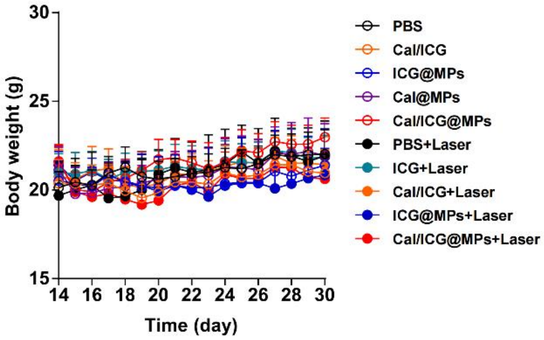

Supplementary Fig. 40 Body weight of stroma-rich H22 tumor-bearing mice after treatment with Cal/ICG@MPs upon 808 nm laser irradiation.

Body weight of stroma-rich H22 tumor-bearing mice (constructed by co-injection of H22 cells and TGF-β-activated skin fibroblasts) after intravenous injection of PBS, ICG, Cal/ICG, ICG@MPs, Cal@MPs or Cal/ICG@MPs derived from H22 cells at the ICG dosage of 8 mg kg^{-1} and Cal dosage of 120 μg kg^{-1} twice every two days, followed with or without 808 nm laser irradiation (1.5 W cm^{-2}, 10 min) at 2 h after the last injection. Data are presented as means ± s.d. (n = 8 mice per group). Source data are provided as a Source Data file.

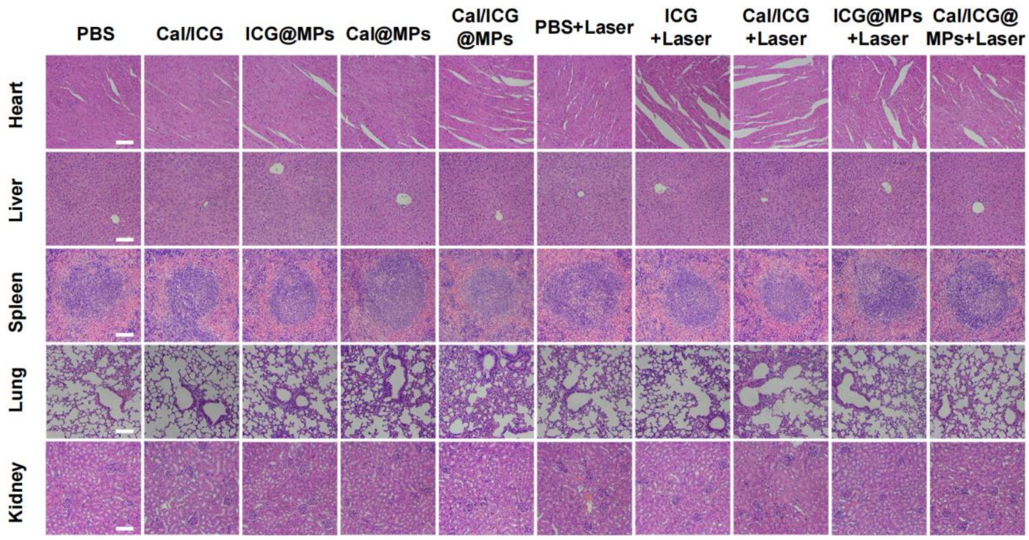

Supplementary Fig. 41 Histological observation of major organs in stroma-rich H22 tumor-bearing mice after treatment with Cal/ICG@MPs upon 808 nm laser irradiation.

Histological observation of major organs in stroma-rich H22 tumor-bearing mice (constructed by co-injection of H22 cells and TGF-β-activated skin fibroblasts) at 14 days after intravenous injection of PBS, ICG, Cal/ICG, ICG@MPs, Cal@MPs or Cal/ICG@MPs derived from H22 cells at the ICG dosage of 8 mg kg^{-1} and Cal dosage of 120 μg kg^{-1} twice every two days, followed with or without 808 nm laser irradiation (1.5 W cm^{-2}, 10 min) at 2 h after the last injection by H&E staining. Images are representative of three biologically independent mice. Scale bars: 50 μm.

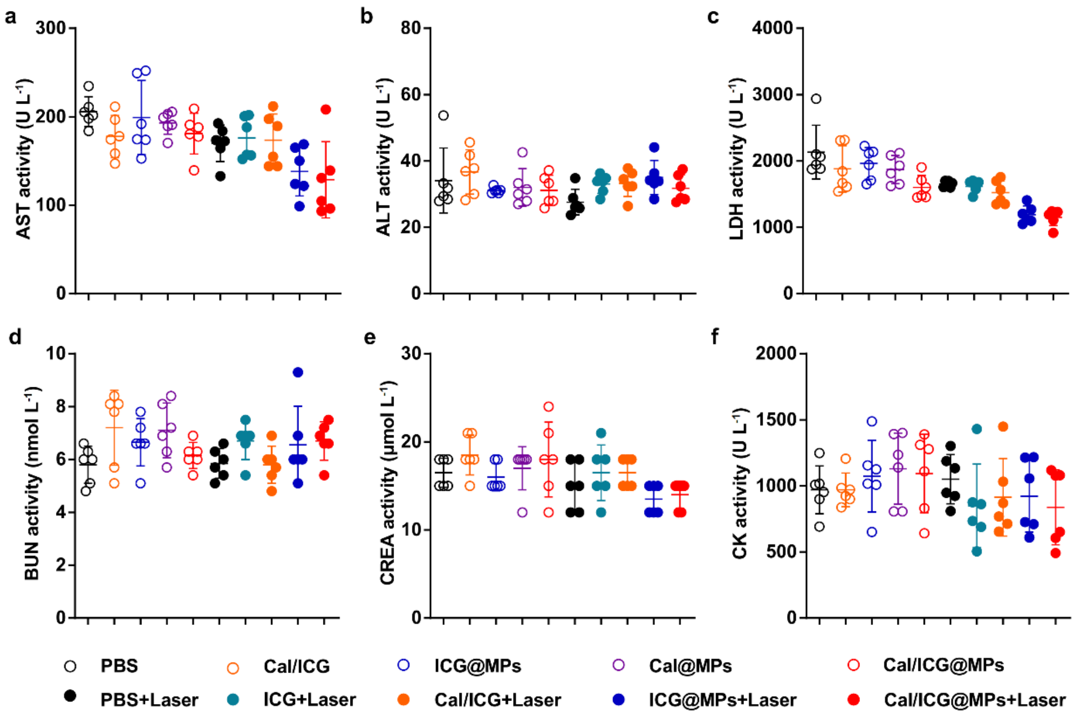

Supplementary Fig. 42 Serological analysis of stroma-rich H22 tumor-bearing mice after treatment with Cal/ICG@MPs upon 808 nm laser irradiation.

a-f Serological analysis of aspartate aminotransferase (AST, **a**), alanine aminotransferase (ALT, **b**), lactate dehydrogenase (LDH, **c**), blood urea nitrogen (BUN, **d**), creatinine (CREA, **e**) and creatine kinase (CK, **f**) in stroma-rich H22 tumor-bearing mice at 14 days after intravenous injection of PBS, ICG, Cal/ICG, ICG@MPs, Cal@MPs or Cal/ICG@MPs derived from H22 cells at the ICG dosage of 8 mg kg^{-1} and Cal dosage of 120 μg kg^{-1} twice every two days, followed with or without 808 nm laser (1.5 W cm^{-2}, 10 min) at 2 h after the last injection. Data are presented as means ± s.d. (n = 6 mice per group). Source data are provided as a Source Data file.

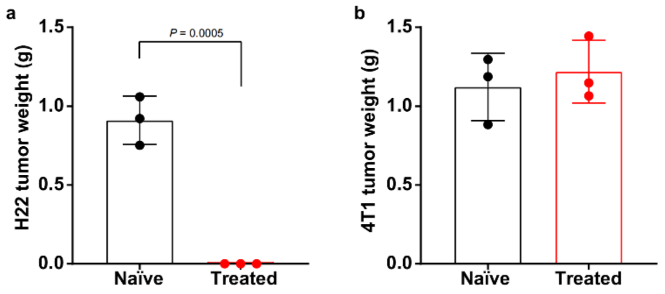

Supplementary Fig. 43 Tumor weight of Cal/ICG@MPs with 808 nm laser irradiation-cured mice undergoing tumor rechallenge.

a, b Tumor weight of rechallenged H22 tumors (**a**) and 4T1 tumors (**b**) in naïve mice or Cal/ICG@MPs with 808 nm laser irradiation-cured mice. Data are presented as means ± s.d. (n = 3 mice per group; two-tailed unpaired t-test). Source data are provided as a Source Data file.

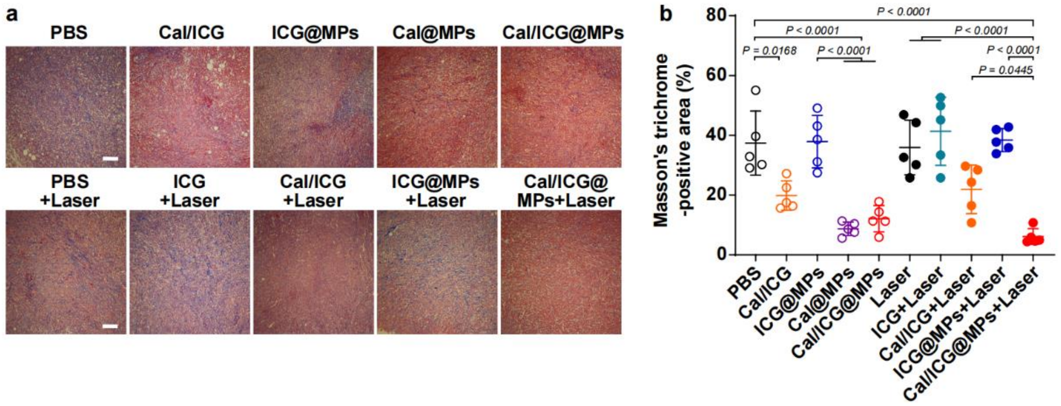

Supplementary Fig. 44 Masson's trichrome staining of primary tumor tissues in stroma-rich 4T1 orthotopic bilateral tumor model upon 808 nm laser irradiation. **a, b** Representative Masson's trichrome staining images (**a**) and quantification of Masson's trichrome staining positive area (**b**) in the primary tumors of stroma-rich 4T1 orthotopic bilateral tumor model after intravenous injection of PBS, ICG, Cal/ICG, ICG@MPs, Cal@MPs or Cal/ICG@MPs derived from 4T1 cells at the ICG dosage of 8 mg kg^{-1} and Cal dosage of 120 μg kg^{-1} twice every two days, followed with or without 808 nm laser irradiation (1.5 W cm^{-2}, 10 min) at 2 h after the last injection. Scale bars: 40 μm. Data are presented as means ± s.d. (n = 5 fields in total from 3 mice; one-way ANOVA followed by Tukey's HSD post-hoc test). Source data are provided as a Source Data file.

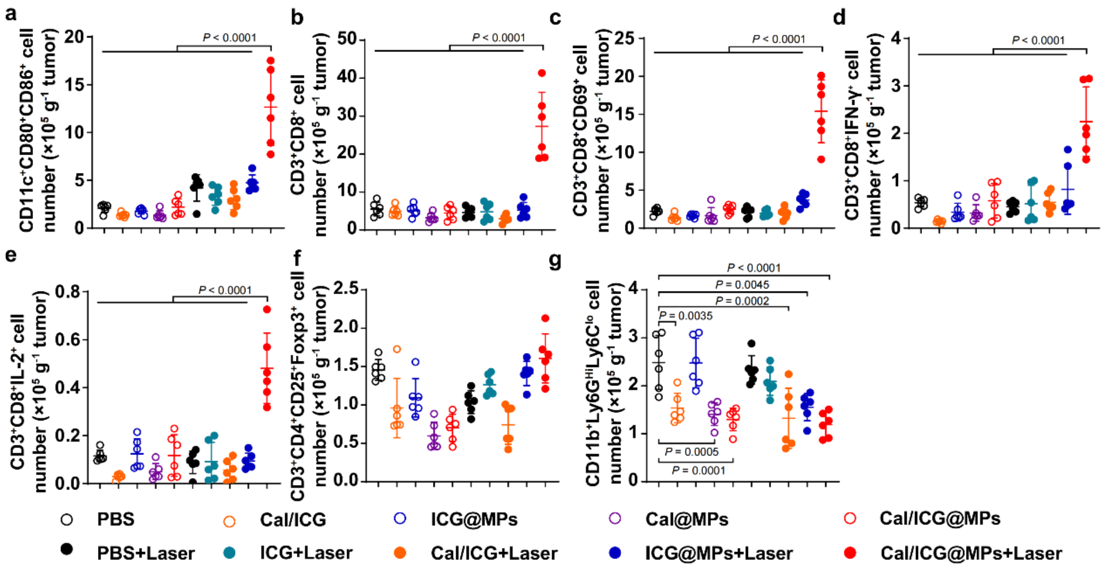

Supplementary Fig. 45 Cal/ICG@MPs-induced improved immune microenvironment in contralateral tumors of stroma-rich 4T1 orthotopic bilateral tumor model upon 808 nm laser irradiation.

a-g Numbers of $CD11c^+CD80^+CD86^+$ cells (**a**), $CD8^+$T cells (**b**), $CD8^+CD69^+$ T cells (**c**), $CD8^+IFN\text{-}\gamma^+$ T cells (**d**), $CD8^+IL\text{-}2^+$ T cells (**e**), Tregs (**f**) and PMN-MDSCs (**g**) in contralateral tumors of stroma-rich 4T1 orthotopic bilateral tumor model after intravenous injection of PBS, ICG, Cal/ICG, ICG@MPs, Cal@MPs or Cal/ICG@MPs derived from 4T1 cells at the ICG dosage of 8 mg kg^{-1} and Cal dosage of 120 μg kg^{-1} twice every two days, followed with or without 808 nm laser irradiation (1.5 W cm^{-2}, 10 min) at 2 h after the last injection. Data are presented as means ± s.d. (n = 6 mice per group; one-way ANOVA followed by Tukey's HSD post-hoc test). Source data are provided as a Source Data file.

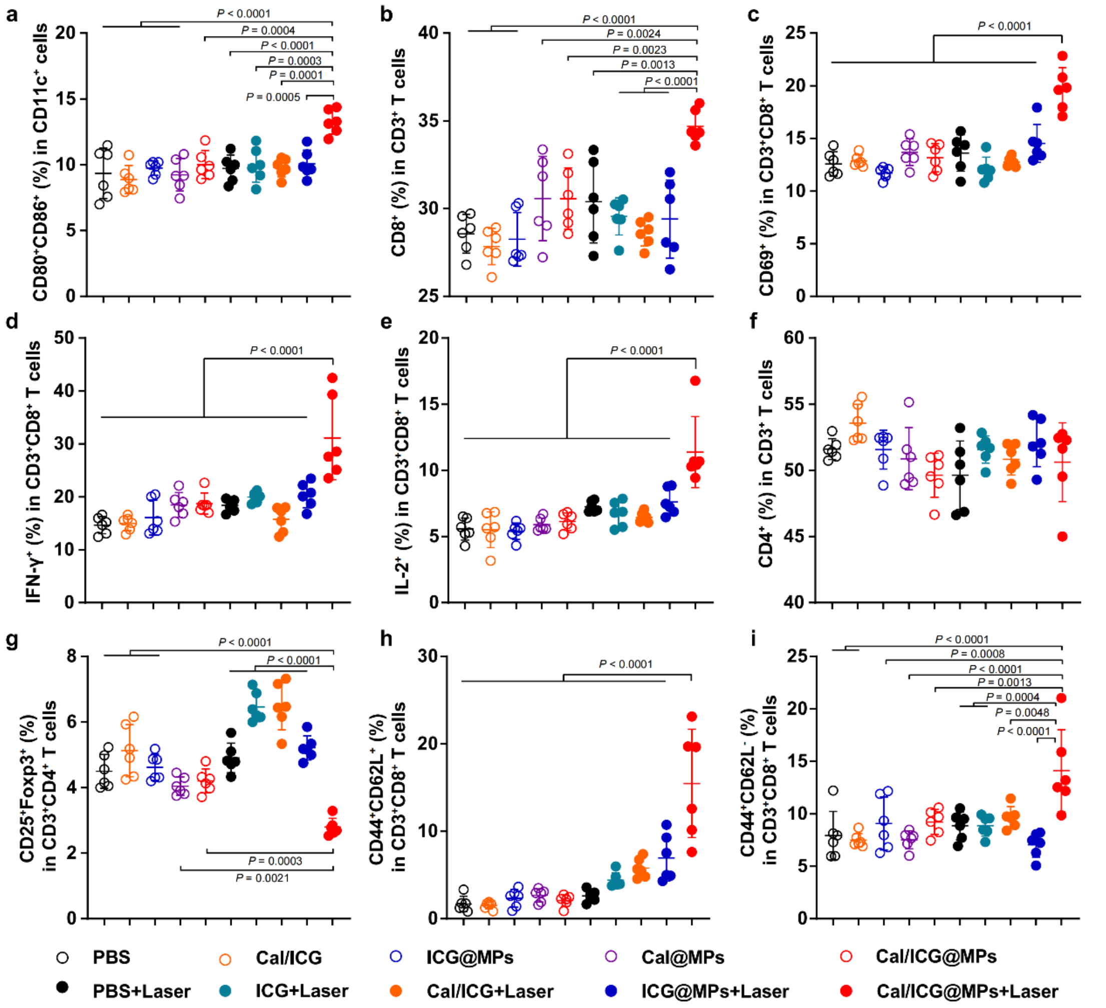

Supplementary Fig. 46 Cal/ICG@MPs-induced improved immune responses in spleens of orthotopic 4T1 tumor-bearing mice upon 808 nm laser irradiation.

a-i Percentages of matured DC (**a**), $CD8^+$ T cells (**b**), $CD8^+CD69^+$ T cells (**c**), $CD8^+IFN\text{-}\gamma^+$ T cells (**d**), $CD8^+IL\text{-}2^+$ T cells (**e**), $CD4^+$ T cells (**f**), Tregs (**g**), $CD8^+$ Tcm cells (**h**) and $CD8^+$ Tem cells (**i**) in spleens of stroma-rich 4T1 orthotopic tumor-bearing mice after intravenous injection of PBS, ICG, Cal/ICG, ICG@MPs, Cal@MPs or Cal/ICG@MPs derived from 4T1cells at the ICG dosage of 8 mg kg^{-1} and Cal dosage of 120 μg kg^{-1} twice every two days, followed with or without 808 nm laser irradiation (1.5 W cm^{-2}, 10 min) at 2 h after the last injection. Data are presented as means ± s.d. (n = 6 mice per group; one-way ANOVA followed by Tukey's HSD post-hoc test). Source data are provided as a Source Data file.

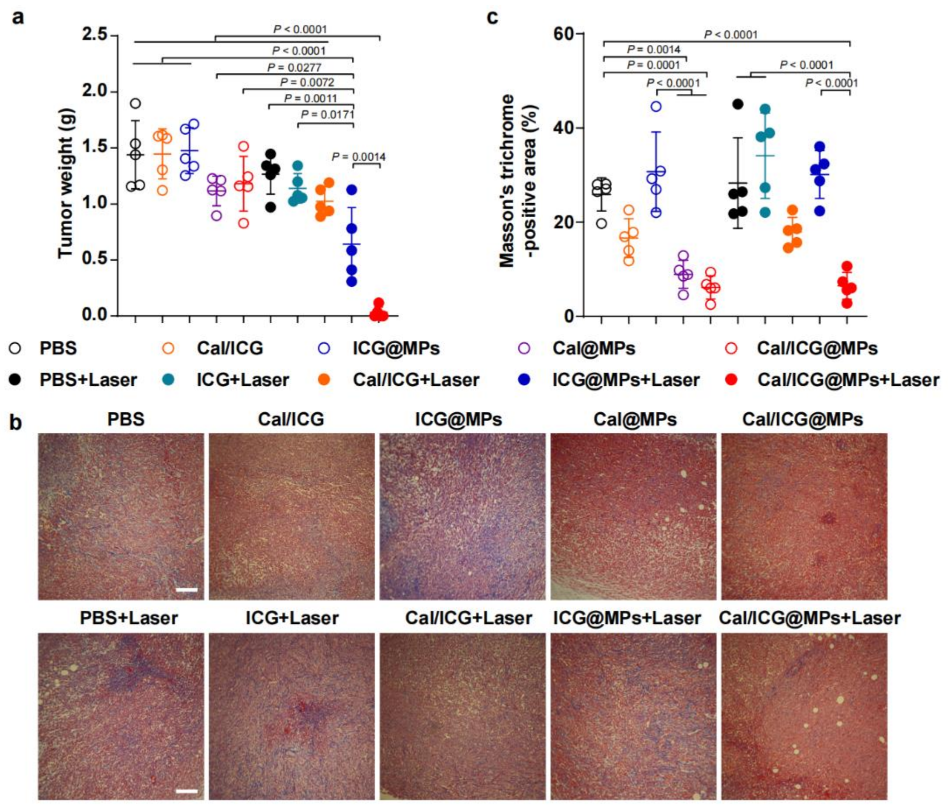

Supplementary Fig. 47 Orthotopic tumor weight and ECM remodeling in 4T1 metastasis tumor models after treatment with Cal/ICG@MPs upon 808 nm laser irradiation.

a Orthotopic tumor weight of 4T1 metastasis tumor models after intravenous injection of PBS, ICG, Cal/ICG, ICG@MPs, Cal@MPs or Cal/ICG@MPs derived from 4T1 cells at the ICG dosage of 8 mg kg^{-1} and Cal dosage of 120 μg kg^{-1} every two days for two times, followed with or without 808 nm laser irradiation (1.5 W cm^{-2}, 10 min) at 2 h after the last injection. Data are presented as means ± s.d. (n = 5 mice per group; one-way ANOVA followed by Tukey's HSD post-hoc test). **b**, **c** Representative Masson's trichrome staining images (**b**) and quantification of Masson's trichrome staining positive area (**c**) in the orthotopic tumors of 4T1 metastasis tumor models after treatment indicated in (**a**). Scale bars: 40 μm. Data are presented as means ± s.d. (n = 5 fields in total from 3 mice; one-way ANOVA followed by Tukey's HSD post-hoc test). Source data are provided as a Source Data file.

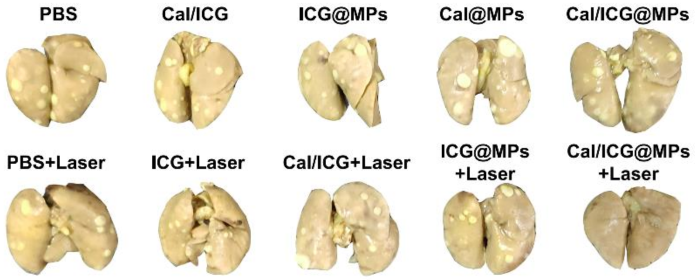

Supplementary Fig. 48 Cal/ICG@MPs-induced metastasis inhibition in 4T1 metastasis tumor models upon 808 nm laser irradiation.

Representative images of the lungs of stroma-rich 4T1 metastasis tumor models after intravenous injection of PBS, ICG, Cal/ICG, ICG@MPs, Cal@MPs or Cal/ICG@MPs derived from 4T1 cells at the ICG dosage of 8 mg kg^{-1} and Cal dosage of 120 μg kg^{-1} twice every two days, followed with or without 808 nm laser irradiation (1.5 W cm^{-2}, 10 min) at 2 h after the last injection indicated in Fig. **9a.** Images are representative of five biologically independent mice.

Supplementary Table 1. ICG loading capacity for ICG@MPs

ICG feeding content ($\mu g\ mL^{-1}$)	ICG loading capacity ($\mu g\ \mu g^{-1}$ protein)
25	0.0635
50	0.1315
100	0.2688
150	0.2835

Supplementary Table 2. Drug loading capacity for Cal/ICG@MPs

ICG feeding content ($\mu g\ mL^{-1}$)	Cal feeding content ($\mu g\ mL^{-1}$)	ICG loading capacity ($\mu g\ \mu g^{-1}$ protein)	Cal loading capacity ($ng\ \mu g^{-1}$ protein)
100	1	0.2730	0.7245
100	2	0.2745	1.5485
100	4	0.2735	3.7129
100	6	0.2732	5.9459
